# Age at onset as an index of genetic heterogeneity in major psychiatric and substance use disorders

**DOI:** 10.1017/S0033291724002630

**Published:** 2024-11

**Authors:** Kenneth S. Kendler, Ohlsson Henrik, Jan Sundquist, Kristina Sundquist

**Affiliations:** 1 Virginia Institute for Psychiatric and Behavioral Genetics, Virginia Commonwealth University, Richmond, VA, USA; 2 Department of Psychiatry, Virginia Commonwealth University, Richmond, VA, USA; 3 Center for Primary Health Care Research, Lund University, Malmö, Sweden; 4 Department of Family Medicine and Community Health, Department of Population Health Science and Policy, Icahn School of Medicine at Mount Sinai, New York, USA

**Keywords:** age at onset, genetic risk, depression, schizophrenia, drug use disorder, genetic heterogeneity

## Abstract

**Background:**

Robust clinical indices of etiologic heterogeneity for psychiatric disorders are rare. We investigate whether age at onset (AAO) reflects genetic heterogeneity, utilizing Genetic Risk Ratios (GRR) derived from Family Genetic Risk Scores (FGRS).

**Methods:**

We examined, in individuals born in Sweden 1940–2003, whether AAO for five primary disorders -- drug use disorder (DUD), alcohol use disorder (AUD), major depression (MD), bipolar disorder (BD), and schizophrenia (SZ)-- was associated with varying levels of GRRs with a range of informative secondary disorders and traits.

**Results:**

Our disorders displayed a varying pattern of change of GRRs with increasing AAO. At one end was SZ, where all GRRs rose with increasing AAO meaning that SZ became increasing genetically heterogeneous with later AAO. The most balanced disorder was AUD where, with increasing AAO, GRRs rose for AD, BD, and MD and declined for DUD, CB, and ADHD. That is, at young AAO, AUD had high levels of genetic risk for other externalizing disorders while at older AAO, high genetic risk for internalizing disorders were more prominent. MD was at the continuum's other end where all GRRs, except for AD, decreased with higher AAO, meaning that MD became increasingly genetically homogeneous with later AAO.

**Conclusions:**

Genetic heterogeneity was robustly associated with AAO across our five primary disorders with substantial inter-disorder differences in the observed patterns. In particular, young AAO was associated with maximal genetic homogeneity for SZ and DUD while older AAO had greater genetic homogeneity for MD with AUD falling in between.

Early in the history of psychiatric genetics, the variable age at onset (AAO) of psychiatric disorders was a nuisance variable that had to be corrected for to obtain accurate risk figures (Scarr, Webber, Weinberg, & Wittig, 1981; Slater & Cowie, 1971). Subsequently, three research questions emerged, each suggesting, in different ways, the possible value of AAO. First, AAO often appeared to be familial, with positive correlations observed in pairs of affected relatives for schizophrenia (SZ) (Kendler, Tsuang, & Hays, 1987; Sham, Zerbin-Rudin, & Kendler, 1995; Zhan, Sham, So, & Lui, 2023), bipolar disorder (BD) (Baron, Mendlewicz, & Klotz, 1981; Lin et al., 2006) and major depression (MD) (Baron et al., 1981; Kendler, Neale, Kessler, Heath, & Eaves, 1992). Second, AAO was found to frequently index familial/genetic risk with numerous studies finding higher risk of illness in relatives of probands with younger *v.* older AAO for SZ (Zhan et al., 2023), MD (Kendler, Gatz, Gardner, & Pedersen, 2005; Lyons et al., 1998; Tozzi et al., 2008; Weissman et al., 1984; Yang et al., 2014), BD (Soni et al., 2021), and alcohol use disorder (AUD) (Buydens-Branch, Branchey, & Noumair, 1989). More recently, molecular genetic risk factors have been shown to influence age of onset of those disorders (Kang et al., 2021; Nurnberger et al., 2022).

The third question, evidence for which has begun to emerge in recent years, is the possibility that AAO might index of etiologic, or more specifically, genetic heterogeneity (Thapar & Riglin, 2020). Lin et al., reported that early AAO in BD was associated with higher risk for drug use disorder (DUD) in relatives, suggesting that ‘age at onset reflects underlying genetic heterogeneity in bipolar disorder (Lin et al., 2006).’ Polygenic risk scores analyses have shown that early-onset MD has a stronger genetic correlation with SZ (Mitchell et al., 2022; Power et al., 2017; Rice et al., 2019) and BD (Musliner et al., 2019; Power et al., 2017) and ADHD (Rice et al., 2019) than later onset MD.

We here seek to expand prior inquiries into AAO as an index of genetic heterogeneity. To do this, we defined a novel genetic construct ‘*Genetic Relative Risk*’ (GRR). We define it as follows: *for a given primary disorder at a given AAO, GRR equals the ratio of the genetic risk for secondary disorders to the genetic risk for the primary disorder*. Assume, for example, that measuring genetic risk on a standardized scale at the AAO of 25, individuals with a primary disorder, say BD, have a mean genetic risk for BD of +0.50 and for a secondary disorder, say MD, of +0.20. Then, for BD at AAO = 25, the GRR of MD equals 0.20/0.50 = 0.40.

In general, geneticists prefer forms of a disorder with low GRRs which reflect a high level of genetic *homogeneity* in that genetic risk for other disorders are making at most modest contributions to the risk for the primary disorder. By contrast, disorders with high GRRs reflect substantial genetic *heterogeneity* in that genetic risks for other disorders are making substantial contributions to risk for the primary disorder. If, as we predict, GRRs for our primary disorders change with AAO, this means that the degree of genetic homogeneity are not fixed features of the disorder but rather vary as a function of their AAO.

We calculate GRRs at AAOs from 15–60 for five primary psychiatric and substance use disorders: AUD, DUD, MD, BD, and SZ on complete samples of patients obtained from Swedish population-based registers. We then evaluate whether AAO is a meaningful index of genetic heterogeneity by examining whether the GRR for 8–12 relevant *secondary* psychiatric disorders and traits change meaningfully as a function of the AAO for these primary disorders.

## Methods

We collected information on individuals from Swedish population-based registers with national coverage linking each person's unique personal identification number which, to preserve confidentiality, was replaced with a serial number by Statistics Sweden. We secured ethical approval for this study from the Regional Ethical Review Board in Lund and no participant consent was required (No. 2008/409 and later amendments).

For our main analysis, we created five separate datasets based on individuals registered for our five primary disorders and calculated age at first registration which was used as a proxy for AAO. Individuals were born between 1940–2003 in Sweden to Swedish born parents. For details of the relevant registers, which includes information from national patient and primary care registers, and diagnostic codes, see appendix tables 1 and 2, respectively. In the datasets, we also included individual family genetic risk scores (FGRS) for the several different disorders, see appendix tables 3. The FGRSs are calculated from morbidity risks for disorders in first-degree through fifth-degree relatives, controlling for cohabitation effects, and thus arise from phenotypes in extended pedigrees, not from molecular genetic data. For details, see appendix table 4.

To examine FGRS profiles at different ages at first registration, we used a linear regression model with the FGRSs as outcome and age at first registration as a continuous variable while controlling for year of birth. We present, in our primary disorders, the predicted FGRS for AAO from ages 15 to 60. Thereafter, we calculated GRRs for each age at first registration, where the denominator was the FGRS for the primary disorder, and the numerator was the FGRSs for the secondary disorders or traits. A core of 7 secondary disorders/traits were examined for all disorders (Anxiety Disorders [AD], ADHD, AUD, BD, DUD, EDU [genetic propensity for *low* educational attainment], and MD) but others were chosen a priori for particular primary individual disorders for their potential importance (e.g., criminal behavior (CB) for AUD and DUD and Autism Spectrum Disorder (ASD) for SZ). We also present the linear slope of all ratios across the 45 years. All analyses were also presented separated by sex. In two sensitivity analyses, we present the ratios based on two separate birth cohorts (1955–64 and 1965–74), and by excluding individuals with relevant comorbidities (see appendix tables 5 and 6). All statistical analyses were performed using SAS 9.4 (SAS Institute, 2012) and R 4.3.1 (R Foundation for Statistical Computing, 2023).

## Results

Table 1 depicts the sample size, sex distribution, and mean AAO for our five primary disorders. Sample sizes ranged from 25 763 cases of SZ to 836 741 cases of AD. BD, MD, and AD had the expected female excess and DUD, AUD, and SZ the expected male excess.

**Table 1.** Number of cases of identified from the Swedish population born 1940–2003 for drug use disorder (DUD), alcohol use disorder (AUD), major depression (MD), bipolar disorder (BD), and schizophrenia (SZ)

	DUD	AUD	SZ	BD	MD^a^
*N*	225 241	332 787	25 763	72 067	806 006
Year of birth (Mean, s.d.)	1975 (16.8)	1961 (15.1)	1959 (13.2)	1969 (16.6)	1970 (17.0)
Males (%)	66.6%	71.6%	60.4%	39.0%	37.0%
Age at first registration (Mean, s.d.)	31.4 (14.3)	38.5 (16.0)	36.6 (13.1)	38.0 (14.0)	40.8 (16.1)
FGRS (Mean, s.d.)	0.663 (1.6)	0.532 (1.4)	0.729 (2.8)	0.616 (1.9)	0.289 (1.1)

a All BD cases are excluded.

**Figure 1.**
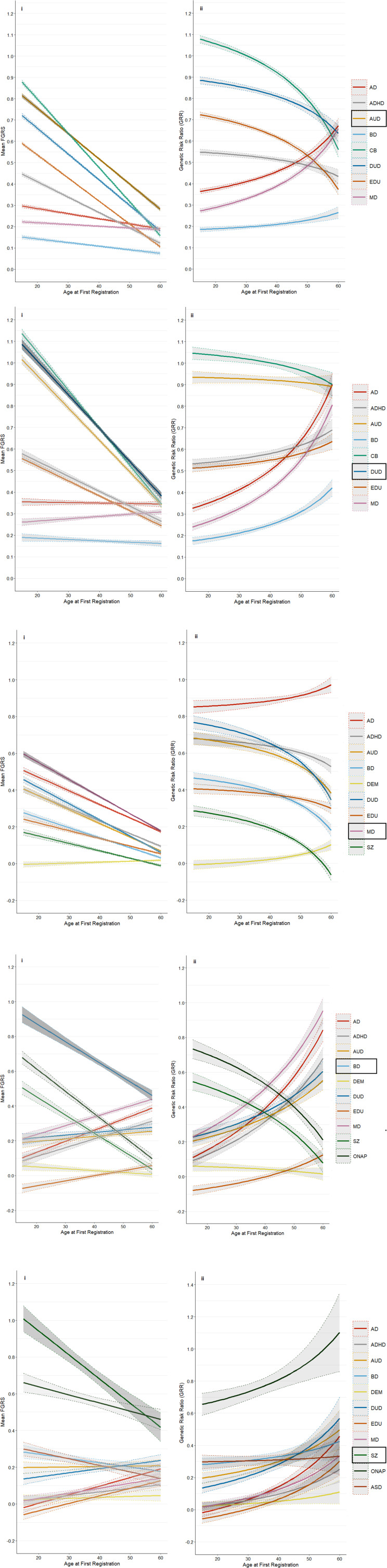
(a) Mean Family Genetic Risk Score (FGRS) (part i) and Genetic Risk Ratio (part ii), both ± 95% CIs, in individuals with alcohol use disorder for ages of onset from 15–60. Part i includes the primary disorder (here alcohol use disorder) with darkened 95% CIs, and the secondary disorders/traits (here Anxiety Disorder (AD), ADHD, bipolar disorder (BD), criminal behavior (CB), drug use disorder (DUD), Low Educational Attainment (EDU), and major depression (MD)). Part ii only includes the secondary disorders/traits. (b) Mean Family Genetic Risk Score (FGRS) (part i) and Genetic Risk Ratio (part ii), both ± 95% CIs, in individuals with drug use disorder for ages of onset from 15–60. Part i includes the primary disorder (here Drug Use Disorder), with darkened 95% CIs, and the secondary disorders/traits (here anxiety disorder (AD)), ADHD, bipolar disorder (BD), criminal behavior (CB), alcohol use disorder (DUD), Low Educational Attainment (EDU), and major depression (MD)). Part ii only includes the secondary disorders/traits. (c) Mean Family Genetic Risk Score (FGRS) (part i) and Genetic Risk Ratio (part ii), both ± 95% CIs, in individuals with major depression for ages of onset from 15–60. Part i includes the primary disorder (here major depression), with darkened 95% CIs, and the secondary disorders/traits (here Anxiety Disorder (AD), ADHD, Alcohol Use Disorder (AUD, Bipolar Disorder (BD), Dementia (DEM), Drug Use Disorder (DUD), Low Educational Attainment (EDU), Major Depression (MD) and Schizophrenia). Part ii only includes the secondary disorders/traits. (d) Mean Family Genetic Risk Score (FGRS) (part i) and Genetic Risk Ratio (part ii), both ± 95% CIs, in individuals with bipolar disorder for ages of onset from 15–60. Part i includes the primary disorder (here bipolar disorder), with darkened 95% CIs, and the secondary disorders/traits (here anxiety disorder (AD), ADHD, alcohol use disorder (AUD), dementia (DEM), drug use disorder (DUD), Low Educational Attainment (EDU), major depression (MD), Schizophrenia and Other Non-Affective Psychosis (ONAP)). (e) Mean Family Genetic Risk Score (FGRS) (part i) and Genetic Risk Ratio (part ii), both ± 95% CIs. in individuals with Schizophrenia. Part i includes the primary disorder (here Schizophrenia (SZ), with darkened 95% CIs, and the secondary disorders/traits (here anxiety disorder (AD), ADHD, alcohol use disorder (AUD), bipolar disorder (BD), dementia (DEM), drug use disorder (DUD), low educational attainment (EDU), major depression (MD), other non-affective psychosis (ONAP) and autism spectrum disorder (AUD)).

The main analyses of our five primary disorders are presented in Figs 1a to 1f. (For individual values at AAO from 15–60 in 5 year intervals, see appendix table 7). The left hand Fig. (i) presents results for the mean FGRS (± 95% CIs) for our secondary disorders/traits in each of our primary disorders as a function of their AAO. The right hand Fig. (ii) takes these results and transforms them into the GRR (± 95% CIs) for each of the secondary disorders/traits. If AAO was unrelated to the genetic heterogeneity of our five primary disorders, than the slope of the GRRs for the secondary disorders should be flat across the AAO distribution of the primary disorders. An inspection of Figs 1aii to 1fii indicates that this is very rarely the case. In almost every instance, the GRRs for the secondary disorders change as a function of the age differences in the onset of the primary disorder. To repeat, given the importance of this primary finding, these results suggest that the relative contribution to the genetic risk for our primary disorders from our secondary disorders and traits almost always vary, and often substantially, with the AAO of the primary disorder. We describe our findings in detail for the first primary disorder – AUD.

Figure 1ai contains eight colored lines each of which represents the mean FGRS for one of the secondary disorders and traits examined in individuals with AUD as a function of their AAO. AUD cases with an onset at age 15, have the highest FGRS for CB and AUD both of which drop steeply with increased AAO. (Looking at the FGRS for the primary disorders in Fig. 1ai to 1ei, all decline with AAO with the slope steepest for DUD and shallowest for MD.) Fig. 1aii contains seven curves of GRRs calculated from the secondary disorder results presented in Fig. 1ai. Taking the gray curve for CB at the top of Fig. 1aii, at AAO of 15 that curve has a GRS value of 1.08 which is obtained by dividing the FGRS_CB_ (equal to 0.88) by the FGRS_AUD_ (equal to 0.81) at that age. All the other curves of GRRs seen in 1aii are derived in a similar way with the numerator and denominator for each AAO equal to, respectively, the FGRS for that disorder/trait and the denominator the FGRS_AUD_.

Turning to the patterns revealed in Fig. 1aii, the seven GRR curves are divisible into two groups. The first is made of up of CB, DUD, EDU, and ADHD where the curves all *decrease* with increasing AAO. That is, *relative to their level of genetic risk for AUD*, the level of genetic risk for CB, DUD and EDU are substantially higher in AUD cases with a young *v.* older AAO. The differences are more modest for ADHD. In the second group of GRRs, we have AD, MD, and BD, which all *increase* with older AAO relative to the level of genetic risk for AUD. For two of these, AD and MD, the increase was substantial with their GRRs more than doubling. So, relative to their level of genetic risk for AUD, the level of genetic risk for AD and MD are substantially higher in AUD cases with an older *v.* younger onset.

For the four other primary disorders, we focus solely on Fig. ii which presents the GRR curves. For DUD (Fig. 1bii), the curves for AD and MD increase steeply with increasing AAO, while for BD, ADHD and EDU, the increase is more modest. By contrast, the GRR for CB and AUD decrease very slowly with increasing AAO.

For MD (Fig. 1cii), six of the eight GRRs declined with increasing AAO (DUD, ADHD, AUD, BD, EDU and SZ) – with increases restricted to AD and Dementia. For BD (Fig. 1dii), we see marked rises in the GRRs for MD, AD, ADHD, AUD, and DUD and modest increases in EDU with higher AAO while the GRRs fall quite strongly for SZ and ONAP and slowly for DEM. Of note, at early AAOs, the values for the GRR for EDU were moderately negative, meaning that BD cases with young AAOs had a high genetic propensity for performing better in school than the general population.

For schizophrenia (Fig. 1eii), the 95% CIs are broad, due to our modest sample size. Aside from the genetic risk for ASD, which was constant across the AAO distribution, all the other GRRs increased with later AAO, led by the ONAP and next most prominent for DUD.

**Figure 2.**
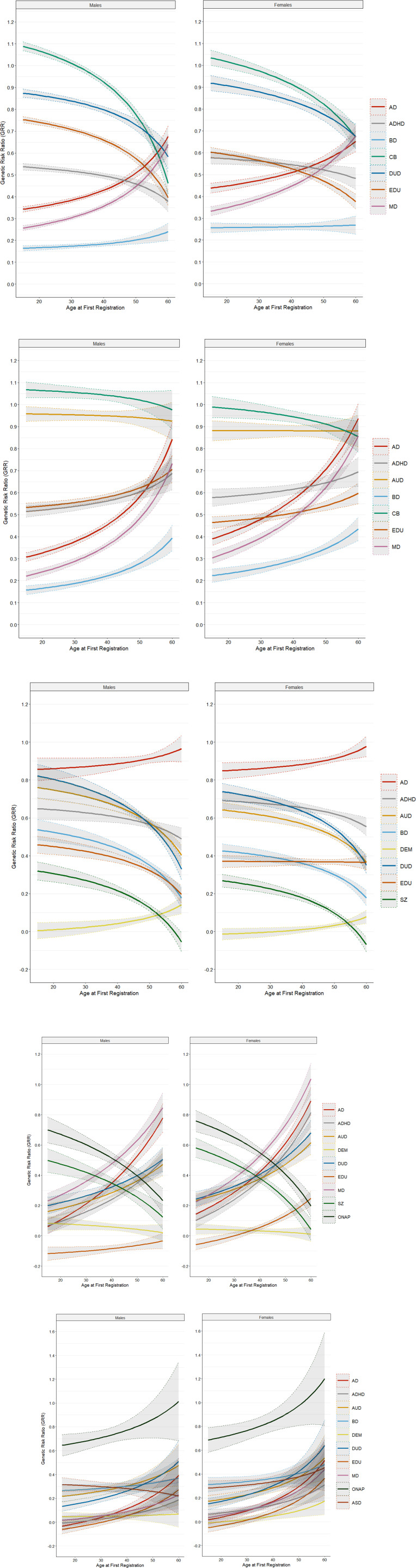
(a) Genetic risk ratios in males and females with alcohol use disorder for ages of onset from 15–60 for all the secondary disorders/traits listed in the legend for Fig. 1a. (b) Genetic risk ratios in males and females with drug use disorder for ages of onset from 15–60 for all the secondary disorders/traits listed in the legend for Fig. 1b. (c) Genetic risk ratios in males and females with major depression for ages of onset from 15–60 for all the secondary disorders/traits listed in the legend for Fig. 1c. (d) Genetic risk ratios in males and females with bipolar disorder for ages of onset from 15–60 for all the secondary disorders/traits listed in the legend for Fig. 1d. (e) Genetic risk ratios in males and females with schizophrenia for ages of onset from 15–60 for all the secondary disorders/traits listed in the legend for Fig. 1e.

We then examine the degree of resemblance for our GRR results as a function of AAO for our five primary disorders separately in men and women (Fig. 2a–e). In general, the overall pattern of GRRs were similar but some differences were observed. Three were noteworthy: (i) in AUD, the GRR for CB falls more rapidly with increasing AAO in males than females, (ii) in DUD, the GRRs for MD and AD are higher and rise more quickly in females than males, and (iii) in MD, the GRR for EDU in males falls with increasing AAO but is stable over varying AAOs in females.

## Discussion

We sought, in these analyses, to explore in greater detail than previously attempted, the relationship between AAO and genetic heterogeneity in major psychiatric and substance use disorders. Of the many findings in our analyses, we emphasize four. *First*, as seen in Figs 1aii–1eii, the GRR is very rarely constant over the AAO of the primary disorder and often changes substantially. This constitutes robust evidence for the hypothesis, supported by prior studies (Mitchell et al., 2022; Musliner et al., 2019; Power et al., 2017; Rice et al., 2019; Thapar & Riglin, 2020), *that AAO meaningfully indexes genetic heterogeneity in major psychiatric disorders.*

**Figure 3.**
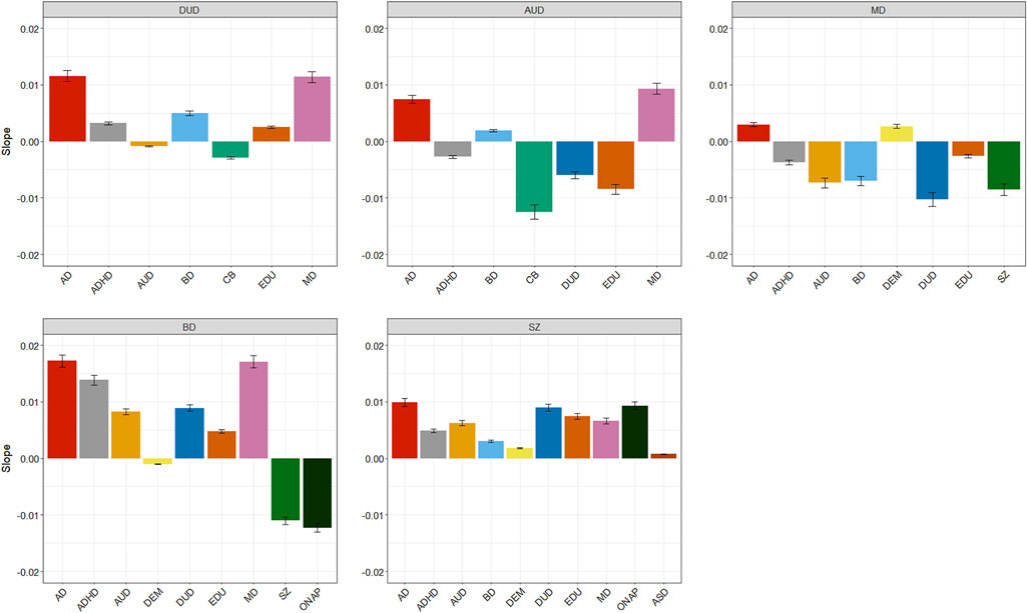
The linear component of the slope of the genetic risk ratios overall age at onsets 15–60 for all secondary disorders for, respectively, drug use disorder (DUD), alcohol use disorder (AUD), major depression (MD), bipolar disorder (BD), and schizophrenia (SZ).

*Second*, as illustrated in Fig. 3, our five primary disorders could be sorted along a continuum of increasing *v.* decreasing genetic heterogeneity with rising AAO. On one end is MD, where, when focusing only on psychiatric and substance use secondary disorders, older AAO saw a modestly larger relative genetic contribution from AD but larger decreasing GRRs for DUD, ADHD, AUD, BD, and SZ. That is, our analyses suggest that older onset forms of MD are more genetically homogeneous than early onset cases. SZ is at the opposite end of the continuum from MD, where the GRR for every secondary psychiatric disorder increases with increasing AAO. In a clear contrast to MD, early onset cases of SZ are more genetically homogenous than cases with older onsets.

DUD was also toward the SZ-end of the continuum with four of five psychiatric disorders (MD, AD, ADHD and BD) have rising GRRs with increasing AAO. BD and AUD were more in this middle. For BD, more psychiatric disorders (MD, AD, ADHD, DUD and AUD) had increasing GRRs with a rising AAO, but GRRs for two disorders – SZ and ONAP – had striking declines. AUD had the most ‘balanced’ picture where with increasing AAO, rising GRRs were seen for three disorders – AD, BD, and MD – and declining GRRs were seen two disorders and one trait – ADHD, DUD, and CB. These results suggest qualitative genetic differences between early and late onset forms of BD and AUD. For BD, genetic contributions to risk were much higher for SZ and ONAP in younger than older onset cases and were much stronger for MD and AD for late *v.* early onset cases. Early onset AUD had strong genetic contributions from three externalizing disorders/traits (ADHD, DUD, and CB) while late onset AUD had rising genetic risks for two classical internalizing disorders: AD and MD.

*Third*, we had two ‘historical/conceptual’ pairs among our primary disorders where our analyses further inform their genetic inter-relationship: AUD/DUD and MD/BD. For both AUD and DUD, meaningful differences were seen. CB had the highest GRR across most of the AAO distribution for both disorders, but its contribution declined much faster for AUD than DUD. While the GRR for ADHD and EDU increased modestly with higher AAO for DUD, the reverse was seen for AUD.

BD and MD both shared two important features – with increasing AAO, the GRR for AD increased and the GRR of SZ sharply decreased. But two differences were also striking. With older AAO, the GRR for MD increased substantially in BD cases while the GRR for BD decreased sharply in MD cases. Also, the GRRs for the externalizing disorders (AUD, DUD, and ADHD) increased with advancing AAO in BD and decreased with older AAO in MD. In broad terms, our results suggest that the most genetically homogenous forms of the two disorders occur on the opposite ends of adult development – early adulthood for BD and large adulthood for MD.

Fourth, we included in some of our analyses, the genetic propensity to three non-psychiatric disorders/traits – CB, EDU, and DEM – because they might reveal other aspects of genetic heterogeneity by AAO. They did not disappoint. Genetic risk to CB contributed strongly to our two substance use disorders. EDU was strongly correlated with risk for AUD and DUD, but this effect declined with increasing AAO in AUD but increased modestly in DUD. Contrary to expectations, the GRR for EDU increased with increasing AAO in SZ and BD, but the maximal levels reached were substantially lower for BD. By contrast, the GRR of EDU decreased in MD with increasing AAO.

We included Dementia as a secondary disorder because of our interest in their possible contribution to late-onset psychiatric disorders such as depression (Brzezińska et al., 2020). Interestingly, the GRR for Dementia increased modestly with older AAO in MD but not BD. SZ had a modest and rising GRR risk for DEM beginning with AAO around 40.

### Limitations

These results should be interpreted in the context of six potential methodological limitations of our analyses. First, the value of our results is dependent upon the validity of the diagnoses obtained from the Swedish registries. These were not diagnoses made in research settings so variability in these clinical conditions is likely. The validities of the hospital diagnoses for SZ and BD in Sweden have, however, been well supported (Ekholm et al., 2005; Lichtenstein et al., 2006; Sellgren, Landen, Lichtenstein, Hultman, & Langstrom, 2011). The validity of the diagnosis of MD and AD are supported by their prevalence, sex ratio, sibling and twin correlations and associations with known psychosocial risk factors (Kendler, Ohlsson, Bacanu, Sundquist, & Sundquist, 2018a, 2018b; Sundquist, Ohlsson, Sundquist, & Kendler, 2017). The validity of our definitions of AUD and DUD is supported by the high rates of concordance across ascertainment methods (Kendler, Lönn, Salvatore, Sundquist, & Sundquist, 2018a, 2018b, 2015) and patterns of resemblance in relatives similar to those found in personally interviewed samples (Prescott & Kendler, 1999; Tsuang et al., 1996). The diagnosis of ADHD in Sweden in validated by its close relationship with the receipt of stimulant medication (Sundquist, Ohlsson, Sundquist, & Kendler, 2015). Second, we do not have precise estimates of AAO in our samples and had to use, instead, ages at first registration. Our estimates of AAO are therefore likely to be, to some extent, upwardly biased.

Third, our analyses focused on the degree of heterogeneity of the genetic risk for our primary disorders, not its magnitude. As clearly seen in Figs 1ai-1ei, the magnitude of genetic risk for all our primary disorders decreased substantially, and at different rates, with rising AAO. That finding, however, is not our focus here.

Fourth, the FGRS, a family phenotype-based measure to assess quantitative genetic risk distinct from polygenic risk scores, has been now widely published, (Kendler, Ohlsson, Sundquist, & Sundquist, 2021a, 2021b,2023a, 2023b, 2023c, 2023d, 2023e) with evidence that it is not highly sensitive to assumptions involved in its calculation, that the correction for cohabitation effects performs appropriately, the method agrees well with other similar statistical approaches (Hujoel, Gazal, Loh, Patterson, & Price, 2020; Krebs et al., 2024). Furthermore, recent empirical analyses and simulations demonstrate that the observed modest correlations between FRGS-like statistics and PRS from the iPsych study for psychiatric disorders are consistent with the hypothesis that current phenotype-based extended family measures, like those used in this study, and molecular based polygene scores are both fallible measures of the same underlying large set of small effect genetic risk alleles (Krebs et al., 2023). Nonetheless, we cannot rule out some biases in the FGRS based, for example, on leakage of shared environment effects or physician bias in the assignment of diagnoses based on their knowledge of the presence of the same disorder in relatives.

Fifth, we did not, in our main analysis, examine the impact of comorbidities in our primary disorders which, in accord with prior studies (Kessler, 1997), are documented in appendix table 8. Therefore, in appendix figures 1a–1e, we explore the impact, on the GRR profiles for our primary disorders, of censoring, from our samples, individuals with particular patterns of comorbidity. Overall, the effects are relatively specific, reducing the GRR only for the eliminated comorbid disorder and in some cases, a closely related disorder. For example, in AUD (appendix figure 1a), censoring from the AUD sample all individuals with a DUD diagnosis – 25% of the sample – reduces only modestly the GRR levels for DUD and increasing the GRR for EDU. Censoring all individuals with MD – 33% of the sample – reduces more appreciably both the GRR levels of MD and AD – and attenuates the risk of the GRR for these two genetics risks with increasing AAO. These results suggest that in individuals with AUD, an appreciable portion of the increasing GRR seen for the genetic risk for MD and AD with increasing AAO occurs in individuals with comorbid MD.

Finally, in addition to exploring the stability of our findings across sex, we also examined their stability across birth cohorts, in appendix Figs 2a to 2e. There we examine, for each primary disorder, the results of our GRR by AAO analyses, using a restricted range of AAOs (20–45) in individuals born in 1955–1964 and 1965–1974. We saw some notable differences between the younger and older cohorts, in particular, higher GRRs from AD and MD in DUD cases, ONAP and SZ in BD cases and AUD and DUD in SZ cases. These changes could arise from alterations in the diagnosis system (ICD-10 was introduced to Sweden in 1997), the functioning of the Swedish registries (e.g. primary care data largely became available after 2000) and/or changes in prevalence (e.g. rising rates of diagnosed ADHD and DUD in Sweden over recent decades Giacobini, Medin, Ahnemark, Russo, & Carlqvist, 2018; Giordano et al., 2014).

## Conclusions

Age of onset is a potent index of levels of genetic heterogeneity in major psychiatric and substance use disorders. Our results argue against the prior dominant concept that the genetic relationship between two psychiatric disorders reflect fixed features of the conditions considered. Rather, the genetic relationship across pairs of disorders demonstrate fluidity as a function of AAO. In a broader sense, these results challenge the hypothesis, dominant in our psychiatric research tradition, that diagnostic categories will capture much of the critical variability that we seek to understand, or the alternative model of diagnostic cynicism that psychiatric assessments are so limited as to be of little value. Rather, they suggest that beneath diagnostic categories are further assessable sources of important variation. We do not need to abandon the use of psychiatric categories, but they likely need deeper supplementary measures that provide important further insights, as we suggest here for AAO.

## Supporting information

Kendler et al. supplementary material Kendler et al. supplementary material

## Supplementary material

The supplementary material for this article can be found at https://doi.org/10.1017/S0033291724002630.

## Access to data and data analysis

Kristina Sundquist MD PhD had full access to all the data in the study and takes responsibility for the integrity of the data and the accuracy of the data analysis.
