# Supplementary material for: Age at onset as an index of genetic heterogeneity in major psychiatric and substance use disorders
Source: Psychol Med. 2024 Dec 2;54(15):4335–47. doi: 10.1017/S0033291724002630 (PMC11650156; doi:10.1017/S0033291724002630)

Appendix

Table 1: **Description of Registers**

*Multi-Generation Register*

The Multi-Generation Register is a register made up of persons who have been registered in Sweden at some time since 1961 and those who were born in 1932 or later. These are called index persons. The register contains connections between index persons and their biological parents. There are about 11 million index persons in the register. The Multi-Generation Register is a part of the register system for Total Population Register, where information comes from the National Tax Board. Every year, a new version of the register is created, including new index persons who immigrated or were born during the year. Information from the Multi-Generation Register may be disclosed for research and statistical purposes. For more information, see *Statistics Sweden, Background Facts, Population and Welfare Statistics 2017:2, Multi-generation register 2016. A description of contents and quality*

*National Patient Register*

In the 1960's the National Board of Health and Welfare started to collect information regarding in-patients at public hospitals, the National Patient Register (NPR). Initially it contained information about all patients treated in psychiatric care and approximately 16 percent of patients in somatic care. The register at that time covered six of the 26 county councils in Sweden. In 1984, the Ministry of Health and Welfare together with the Federation of County Councils decided a mandatory participation for all county councils. From 1987, NPR includes all in-patient care in Sweden. Since 2001, the register also covers outpatient doctor visits including day surgery and psychiatric care from both private and public caregivers. For more information, see *https://www.socialstyrelsen.se/en/statistics-and-data/registers/register-information/the-national-patient-register/*

*Primary Care Data*

We also used information from Primary Care. This is a research dataset including individual-level information on clinical diagnoses from primary health care centers. In the end of the follow-up period the registers covers almost 100% of the population. Figure 1 below show the percentage of the entire Swedish population that resides in counties with primary care data. For more information see: Sundquist, J., Ohlsson, H., Sundquist, K., Kendler, KS. Common adult psychiatric disorders in Swedish primary care where most mental health patients are treated. BMC Psychiatry 17, 235 (2017). <https://doi.org/10.1186/s12888-017-1381-4>

Percentage of the entire Swedish population that resides in counties with primary care data by Year

*
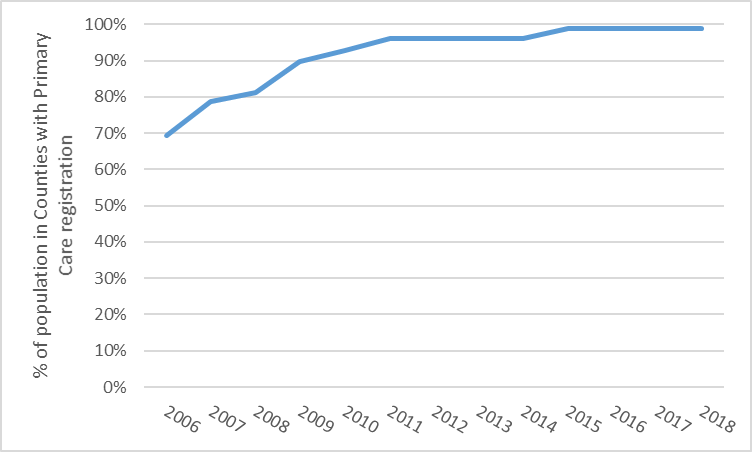
*

Table 2: **Definition of Variables**

|  | Registers Used | Definition |
| --- | --- | --- |
| Schizophrenia (SZ) | The Swedish Hospital Discharge Register (coverage 1997-2018); Outpatient Care Register (national coverage 2001-2018); Primary Care Registry (Partly coverage from 1999-2018) | ICD-8: 295.1, 295.2, 2953, 295.9, 295.6; ICD-9: 295B, 295C, 295D, 295G, 295X; ICD-10: F200, F201, F202, F203, F205, F209 |
| Major Depression (MD) | The Swedish Hospital Discharge Register (coverage 1973-2018); Outpatient Care Register (national coverage 2001-2018); Primary Care Registry (Partly coverage from 1999-2018) | ICD-8: 296.2, 298.0, 300.4; ICD-9: 296.2, 296.4, 298.0, 300.4; ICD-10: F32, F33. **Note that individuals with a BD registration are excluded** |
| Bipolar Disorder (BD) | The Swedish Hospital Discharge Register (coverage 1973-2018); Outpatient Care Register (national coverage 2001-2018); Primary Care Registry (Partly coverage from 1999-2018) | ICD-8: 296.1, 296.3, 296.8, 296.9, 298.1; ICD-9: 296A, 296C, 296D, 296E, 296W, 298B; ICD-10: F30, F31 |
| Drug Use Disorder (DUD) | The Swedish Hospital Discharge Register (coverage 1973-2018); Outpatient Care Register (national coverage 2001-2018); Primary Care Registry (Partly coverage from 1999-2018); The National Prescribed Drug Register (2005-2018); the Swedish Mortality Register, and the Swedish Criminal Register (1973-2018) and the Swedish Suspicion Register (1998-2018) | DUD was identified in the Swedish medical and mortality registries by ICD codes (ICD8: Drug dependence (304); ICD9: Drug psychoses (292) and Drug dependence (304); ICD10: Mental and behavioral disorders due to psychoactive substance use (F10-F19), except those due to alcohol (F10) or tobacco (F17)); in the Suspicion Register by codes 3070, 5010, 5011, and 5012, that reflect crimes related to DA; and in the Crime Register by references to laws covering narcotics (law 1968:64, paragraph 1, point 6) and drug-related driving offences (law 1951:649, paragraph 4, subsection 2 and paragraph 4A, subsection 2). DA was identified in individuals (excluding those suffering from cancer) in the Prescribed Drug Register who had retrieved (in average) more than four defined daily doses a day for 12 months from either of Hypnotics and Sedatives (Anatomical Therapeutic Chemical (ATC) Classification System N05C and N05BA) or Opioids (ATC: N02A). |
| Alcohol Use Disorder (AUD) | The Swedish Hospital Discharge Register (coverage 1973-2018); Outpatient Care Register (national coverage 2001-2018); Primary Care Registry (Partly coverage from 1999-2018); the Swedish Drug Register (2005-2018); the Swedish Mortality Register, and the Swedish Criminal Register (1973-2018) and the Swedish Suspicion Register (1998-2018) | Alcohol Use Disorder (AUD) was identified in the Swedish medical and mortality registries by ICD codes: ICD9: V79B, 305A, 357F, 571A-D, 425F, 535D, 291, 303, 980; ICD 10: E244, G312, G621, G721, I426, K292, K70, K852, K860, O354, T51, F10); in the Crime Register by codes 3005, 3201, which reflect crimes related to alcohol abuse; in the Suspicion Register by codes 0004, 0005 (Only those individuals with at least two alcohol-related crimes or suspicion of crimes from both Crime Register and Suspicion Register were included); in the Prescribed Drug Register by the drugs disulfiram (Anatomical Therapeutic Chemical (ATC) Classification System N07BB01), acamprosate (N07BB03), and naltrexone (N07BB04). |
|  |  |  |
| ADHD | The Swedish Hospital Discharge Register (coverage 1973-2017); Outpatient Care Register (national coverage 2001-2017); Primary Care Registry (Partly coverage from 1999-2017) | ICD-9: 314; ICD-10: F90 |
| Autism spectrum disorder (ASD) | The Swedish Hospital Discharge Register (coverage 1973-2017); Outpatient Care Register (national coverage 2001-2017); Primary Care Registry (Partly coverage from 1999-2017) | ICD-9: 299; ICD-10: F840, F841, F845, F849 |
| Anxiety Disorder (AD) | The Swedish Hospital Discharge Register (coverage 1973-2018); Outpatient Care Register (national coverage 2001-2018); Primary Care Registry (Partly coverage from 1999-2018) | ICD-8: 300.0, 300.2; ICD-9: 300A, 300C; ICD-10: F40, F41 |
| Other Non-affective psychosis (ONAP) | The Swedish Hospital Discharge Register (coverage 1973-2018); Outpatient Care Register (national coverage 2001-2018); Primary Care Registry (Partly coverage from 1999-2018) | ICD-8: 297, 298.3, 298.9, 295.4, 295.7; ICD-9: 298E, 298W, 298X, 295E, 295H, 295W; ICD-10: F22, F23, F24, F25, F26, F27, F28, F29, F208. |
| Criminal behavior (CB) | Swedish Crime register (1973-2018) | Excluding convictions for minor crimes like traffic violations. |
| Low Education (EDU) | The longitudinal integration database for health insurance and labor market studies (LISA) from 1990-2018 and The Swedish Census from 1970. | Highest achieved education measured in 1-7 levels  1-Pre-high school (7 years)  2-High School (9 years)  3-Upper Secondary School (11 years)  4-Upper Secondary School (12 years)  5-Post-secondary education (14 years)  6-Post-secondary education (17 years)  7- PhD education (21 years) |
| Dementia (DEM) | The Swedish Hospital Discharge Register (coverage 1973-2018); Outpatient Care Register (national coverage 2001-2018); Primary Care Registry (Partly coverage from 1999-2018) | ICD-8: 290.0, 290.1  ICD-9: 290A, 290B, 290C, 290D, 290E, 331A  ICD-10: F00, F01, F02, F03, G30 |

Table 3**: Definition of the 5 databases**

| Database | Inclusion criteria | Included FGRSs | Denominator in the Ratios | Number of FGRS ratios |
| --- | --- | --- | --- | --- |
| 1. (AUD) | All individuals born between 1940 and 2003 (in Sweden to Swedish born parents) with an AUD registration | AD, ADHD, AUD, BD, CB, DUD, EDU, MD | AUD FGRS | 7 |
| 2. (BD) | All individuals born between 1940 and 2003 (in Sweden to Swedish born parents) with a BD registration | AD, ADHD, AUD, BD, DEM, DUD, EDU, MD, SZ, ONAP | BD FGRS | 9 |
| 3. (DUD) | All individuals born between 1940 and 2003 (in Sweden to Swedish born parents) with a DUD registration | AD, ADHD, AUD, BD, CB, DUD, EDU, MD | DUD FGRS | 7 |
| 4. (MD) | All individuals born between 1940 and 2003 (in Sweden to Swedish born parents) with a MD registration | AD, ADHD, AUD, BD, DEM, DUD, EDU, MD, SZ | MD FGRS | 8 |
| 5 (SZ) | All individuals born between 1940 and 2003 (in Sweden to Swedish born parents) with a SZ registration | AD, ADHD, AUD, BD, DEM, DUD, EDU, MD, SZ, ONAP, ASD | SZ FGRS | 10 |

Table 4: **Calculation of the Familial Genetic Risk Score (FGRS)**

|  |
| --- |
| The dataset for the calculations includes:  Column1 = Identification number of the proband (Born 1932-1995)  Column2 = Identification number of the relative (1st to 5th degree relatives)  Column3 = Proportion of shared additive genetic effects (0.03125 to 0.50) with the proband  Column4 = Year of Birth of relative  Column5 = Sex of relative  Column6 = Age at registration for trait  Column7 = Age at end of follow-up (2018-12-31 or age at death, or age at emigration whichever came first) |
| Step 1: Using all unique relatives with a registration for the disorder, we non-parametrically estimated the distribution of Age at first registration. The empirical distribution is used to obtain weights for relatives without a registration for the disorder, in order to account for the proportion of the time-at-risk period they had completed at the end of follow-up. For example, for relatives at age x at end of follow-up, the weight corresponds to the proportion of relatives registered for the trait that had been registration at age x. For relatives born prior to 1958 we subtracted age at the end of follow-up with the following formula: 1958 - Year of birth of relative. This modification was done in order to control for registration effects (i.e, most registers in Sweden start in 1973 suggesting that relatives from early birth cohorts do not have the possibility to be registered at younger ages). Note that all relatives with the disorder are weighted one. |
| Step 2: Transform the binary variable (trait yes/no) into a z-score based on the threshold for each trait. The underlying liability of the individual is not assessable. Instead we estimated the mean of the underlying liability to obtain sex and birth decade specific Z-scores for relatives with the trait registration and relatives without the trait. We generate n random numbers from a N(0, 1) distribution and estimate the mean for relatives registered with the disorder (i.e., mean of the observations above the threshold) and for relatives without a registration (i.e., mean of all observation below the threshold). The thresholds are calculated for each decade of birth and sex. |
| Step 3: Correct for cohabitation effects. To estimate the cohabitation effect (i.e. “shared environment”), we created a database with all individuals in the Swedish population born in Sweden 1955-1990. We also included the number of years, during ages 0-15, that individuals resided in the same household as their biological father. We thereby were able to define two kinds of families i) “not-lived-with” father families (offspring never resided for more than 1 year in the same household or in the same community as their biological father); ii) “lived-with” father (offspring resided a minimum of 13 year in the same household as their biological father. We performed a logistic regression model with the binary trait in offspring as outcome and the binary trait in father, type of father, and their interaction as predictors. We used the interaction term as the difference of effect between genes only and genes + environment. The same approach was performed for half-siblings where we compared those who were reared together versus reared apart. The following interaction terms were used in the calculations for each of our disorders:   \|  \| Parent/Children \| Siblings \| \| --- \| --- \| --- \| \| MD \| 0.80 \| 0.85 \| \| AD \| 0.87 \| 0.81 \| \| BD \| 0.67 \| 0.77 \| \| SZ \| 0.93 \| 0.84 \| \| AUD \| 0.99 \| 0.69 \| \| DUD \| 0.92 \| 0.52 \| \| ADHD \| 0.42 \| 0.81 \| \| ASD \| 0.83 \| 0.61 \| \| DEM \| 0.95 \| 0.99 \| \| EDU \| 0.82 \| 0.73 \| \| ONAP \| 0.99 \| 0.83 \| \| CB \| 0.94 \| 0.82 \| \|  \| \| \| |
| Step 4: Calculate the product for each relative using the four components:   1. Z-score (reflecting sex and year of birth adjusted rates) 2. Weight (reflecting the proportion of risk period they had completed) 3. Cohabitation effects 4. Proportion of shared genetic effects (0.03125 – 0.5) with the proband |
| Step 5: Average the product calculated in step 4 across all relatives to a proband |
| Step 6: Correct for the number of relatives. We multiplied the results from step 5 with a shrinkage factor. Shrinkage factor (SF): B/(B+A/C). It produces more shrinkage if B and C are small and A is large.   1. the variance of the z-score of the disorder across all relatives, 2. the variance in the mean z-score across all probands, 3. the weighted number of relatives for each proband (sum of Column 3 across each proband). |
| Step 7: Correct for difference by year of birth and county differences. There are 21 counties in Sweden. For each proband we used the county they had resided in during the maximum number of years (measured from 1969 and onwards) We standardized the risk score by year of birth and county of the proband into a z-score with mean 0 and SD 1. |

Table 5: **Sensitivity analysis**

| Database | Sensitivity sample | Exclusion criteria for the sensitivity analyses | %the original sample included in the sensitivity analysis |
| --- | --- | --- | --- |
| 1. (AUD) | A | All individuals with a DUD registration | 74.1% |
|  | B | All individuals with a MD registration | 66.7% |
| 2. (BD) | A | All individuals with a SZ registration as defined in table 6 | 97.7% |
|  | B | All individuals with a DUD registration | 76.4% |
| 3. (DUD) | A | All individuals with an AUD registration | 61.9% |
|  | B | All individuals with a MD registration | 59.0% |
| 4. (MD) | A | All individuals with a DUD registration | 90.0% |
| 5 (SZ) | A | All individuals with a BD registration as defined in table 6 | 91.2% |

Table 6: **Decision Table for BD vs SZ**

In the sensitivity analyses we separated individuals who had both registrations for BD and for SZ. The table below illustrates how individuals were categorized as registered either for BP or for SZ.

|  | | | | | | |
| --- | --- | --- | --- | --- | --- | --- |
|  |  | Number of lifetime SZ diagnoses in the registers | | | | |
|  |  | 1  (Group 1) | 2  (Group 2) | 3-5 (Group 3) | 6-10 (Group 4) | > 10  (Group 5) |
| Number of lifetime BP diagnoses in the registers | 1 (Group 1) | Last diagnosis | Last diagnosis | Most common diagnosis | Most common diagnosis | Most common diagnosis |
|  | 2 (Group 2) | Last diagnosis | Majority of last 3 diagnoses | Majority of last 3 diagnoses | Most common diagnosis | Most common diagnosis |
|  | 3-5 (Group 3) | Most common diagnosis | Majority of last 3 diagnoses | Majority of last 3 diagnoses | Majority of last 3 diagnoses | Majority of last 5 diagnoses |
|  | 6-10 (Group 4) | Most common diagnosis | Most common diagnosis | Majority of last 3 diagnoses | Majority of last 5 diagnoses | Majority of last 5 diagnoses |
|  | > 10 (Group 5) | Most common diagnosis | Most common diagnosis | Majority of last 5 diagnoses | Majority of last 5 diagnoses | Majority of last 5 diagnoses |

Appendix table 7a DUD:

i (mean FGRS)

| Age | AD | ADHD | AUD | BD | CB | DUD | EDU | MD |
| --- | --- | --- | --- | --- | --- | --- | --- | --- |
| 15 | 0.35 (0.34;0.37) | 0.58 (0.56;0.6) | 1.02 (1;1.04) | 0.19 (0.17;0.21) | 1.14 (1.12;1.16) | 1.09 (1.06;1.11) | 0.56 (0.54;0.57) | 0.26 (0.24;0.28) |
| 20 | 0.35 (0.34;0.37) | 0.54 (0.53;0.56) | 0.94 (0.92;0.96) | 0.19 (0.17;0.20) | 1.05 (1.03;1.07) | 1.01 (0.99;1.03) | 0.52 (0.51;0.53) | 0.27 (0.25;0.28) |
| 25 | 0.35 (0.34;0.36) | 0.51 (0.49;0.52) | 0.87 (0.85;0.88) | 0.18 (0.17;0.20) | 0.96 (0.95;0.97) | 0.93 (0.91;0.95) | 0.49 (0.48;0.50) | 0.27 (0.26;0.28) |
| 30 | 0.35 (0.34;0.36) | 0.47 (0.46;0.48) | 0.79 (0.78;0.80) | 0.18 (0.17;0.19) | 0.87 (0.86;0.88) | 0.85 (0.84;0.86) | 0.45 (0.44;0.46) | 0.28 (0.27;0.29) |
| 35 | 0.35 (0.34;0.36) | 0.44 (0.43;0.45) | 0.72 (0.71;0.72) | 0.18 (0.17;0.19) | 0.78 (0.78;0.79) | 0.77 (0.76;0.78) | 0.42 (0.41;0.42) | 0.28 (0.27;0.29) |
| 40 | 0.35 (0.34;0.36) | 0.40 (0.40;0.41) | 0.64 (0.63;0.65) | 0.17 (0.17;0.18) | 0.70 (0.69;0.70) | 0.70 (0.69;0.70) | 0.38 (0.38;0.39) | 0.29 (0.28;0.29) |
| 45 | 0.35 (0.34;0.35) | 0.37 (0.36;0.38) | 0.57 (0.56;0.57) | 0.17 (0.16;0.18) | 0.61 (0.60;0.62) | 0.62 (0.61;0.63) | 0.35 (0.34;0.35) | 0.29 (0.29;0.30) |
| 50 | 0.35 (0.34;0.35) | 0.33 (0.32;0.34) | 0.49 (0.48;0.50) | 0.17 (0.16;0.18) | 0.52 (0.51;0.53) | 0.54 (0.53;0.55) | 0.31 (0.31;0.32) | 0.30 (0.29;0.31) |
| 55 | 0.35 (0.34;0.35) | 0.3 (0.29;0.31) | 0.42 (0.40;0.43) | 0.16 (0.15;0.17) | 0.43 (0.42;0.44) | 0.46 (0.45;0.47) | 0.28 (0.27;0.29) | 0.30 (0.29;0.31) |
| 60 | 0.34 (0.33;0.36) | 0.26 (0.25;0.28) | 0.34 (0.33;0.36) | 0.16 (0.15;0.17) | 0.34 (0.33;0.36) | 0.38 (0.37;0.4) | 0.24 (0.23;0.25) | 0.31 (0.30;0.32) |

ii (Genetic Risk Ratio (GRR))

| Age | AD | ADHD | AUD | BD | CB | EDU | MD |
| --- | --- | --- | --- | --- | --- | --- | --- |
| 15 | 0.33 (0.31; 0.34) | 0.53 (0.51; 0.55) | 0.93 (0.91; 0.96) | 0.17 (0.16; 0.19) | 1.05 (1.02; 1.07) | 0.51 (0.50; 0.53) | 0.24 (0.22; 0.26) |
| 20 | 0.35 (0.34; 0.37) | 0.54 (0.52; 0.56) | 0.93 (0.91; 0.96) | 0.18 (0.17; 0.20) | 1.04 (1.01; 1.07) | 0.52 (0.50; 0.53) | 0.26 (0.25; 0.28) |
| 25 | 0.38 (0.37; 0.39) | 0.55 (0.53; 0.56) | 0.93 (0.91; 0.95) | 0.20 (0.18; 0.21) | 1.03 (1.01; 1.06) | 0.52 (0.51; 0.54) | 0.29 (0.28; 0.30) |
| 30 | 0.41 (0.40; 0.42) | 0.56 (0.54; 0.57) | 0.93 (0.91; 0.95) | 0.21 (0.20; 0.22) | 1.02 (1.00; 1.04) | 0.53 (0.52; 0.54) | 0.32 (0.31; 0.34) |
| 35 | 0.45 (0.44 ;0.46) | 0.57 (0.55; 0.58) | 0.93 (0.91; 0.94) | 0.23 (0.22; 0.24) | 1.01 (1.00; 1.03) | 0.54 (0.53; 0.55) | 0.36 (0.35; 0.37) |
| 40 | 0.50 (0.49 ;0.51) | 0.58 (0.57; 0.59) | 0.92 (0.91; 0.94) | 0.25 (0.24; 0.26) | 1.00 (0.98; 1.02) | 0.55 (0.54; 0.56) | 0.41 (0.40; 0.42) |
| 45 | 0.56 (0.55; 0.58) | 0.60 (0.58; 0.61) | 0.92 (0.90; 0.94) | 0.28 (0.26; 0.29) | 0.99 (0.97; 1.01) | 0.56 (0.55; 0.58) | 0.47 (0.46; 0.49) |
| 50 | 0.64 (0.62; 0.66) | 0.62 (0.60; 0.64) | 0.91 (0.89; 0.94) | 0.31 (0.29; 0.33) | 0.97 (0.94; 0.99) | 0.58 (0.56; 0.60) | 0.55 (0.54; 0.57) |
| 55 | 0.75 (0.72; 0.78) | 0.65 (0.62; 0.68) | 0.90 (0.87; 0.94) | 0.36 (0.33; 0.38) | 0.94 (0.90; 0.98) | 0.60 (0.58; 0.63) | 0.66 (0.63; 0.69) |
| 60 | 0.90 (0.85; 0.95) | 0.69 (0.64; 0.74) | 0.89 (0.84; 0.95) | 0.42 (0.38; 0.46) | 0.90 (0.85; 0.96) | 0.64 (0.60; 0.67) | 0.81 (0.76; 0.85) |

Appendix table 7b AUD:

i (mean FGRS)

| Age | AD | ADHD | AUD | BD | CB | DUD | EDU | MD |
| --- | --- | --- | --- | --- | --- | --- | --- | --- |
| 15 | 0.30 (0.29; 0.30) | 0.45 (0.44; 0.46) | 0.81 (0.80; 0.82) | 0.15 (0.14; 0.16) | 0.88 (0.87; 0.89) | 0.72 (0.71; 0.73) | 0.59 (0.58; 0.60) | 0.22 (0.21; 0.23) |
| 20 | 0.28 (0.28; 0.29) | 0.41 (0.40; 0.42) | 0.76 (0.75; 0.76) | 0.14 (0.14; 0.15) | 0.80 (0.79; 0.81) | 0.66 (0.65; 0.67) | 0.54 (0.53; 0.54) | 0.22 (0.21; 0.22) |
| 25 | 0.27 (0.27; 0.28) | 0.37 (0.37; 0.38) | 0.70 (0.69; 0.70) | 0.13 (0.13; 0.14) | 0.72 (0.71; 0.72) | 0.60 (0.59; 0.61) | 0.48 (0.48; 0.49) | 0.21 (0.21; 0.22) |
| 30 | 0.26 (0.26; 0.27) | 0.34 (0.33; 0.34) | 0.64 (0.63; 0.64) | 0.13 (0.12; 0.13) | 0.64 (0.63; 0.64) | 0.54 (0.53; 0.55) | 0.43 (0.42; 0.43) | 0.21 (0.21; 0.21) |
| 35 | 0.25 (0.25; 0.25) | 0.30 (0.30; 0.31) | 0.58 (0.57; 0.58) | 0.12 (0.11; 0.12) | 0.56 (0.55; 0.56) | 0.48 (0.48; 0.49) | 0.37 (0.37; 0.38) | 0.21 (0.20; 0.21) |
| 40 | 0.24 (0.23; 0.24) | 0.27 (0.26; 0.27) | 0.52 (0.51; 0.52) | 0.11 (0.10; 0.11) | 0.48 (0.47; 0.48) | 0.42 (0.42; 0.43) | 0.32 (0.32; 0.32) | 0.20 (0.20; 0.21) |
| 45 | 0.23 (0.22; 0.23) | 0.23 (0.23; 0.24) | 0.46 (0.45; 0.46) | 0.10 (0.10; 0.10) | 0.40 (0.39; 0.40) | 0.36 (0.35; 0.37) | 0.27 (0.26; 0.27) | 0.20 (0.19; 0.20) |
| 50 | 0.21 (0.21; 0.22) | 0.19 (0.19; 0.20) | 0.40 (0.39; 0.41) | 0.09 (0.09; 0.10) | 0.32 (0.31; 0.32) | 0.30 (0.29; 0.31) | 0.21 (0.21; 0.22) | 0.19 (0.19; 0.20) |
| 55 | 0.20 (0.20; 0.21) | 0.16 (0.15; 0.17) | 0.34 (0.33; 0.35) | 0.08 (0.08; 0.09) | 0.24 (0.23; 0.24) | 0.24 (0.23; 0.25) | 0.16 (0.15; 0.16) | 0.19 (0.18; 0.20) |
| 60 | 0.19 (0.18; 0.20) | 0.12 (0.11; 0.13) | 0.28 (0.27; 0.29) | 0.07 (0.07; 0.08) | 0.16 (0.15; 0.17) | 0.18 (0.17; 0.19) | 0.10 (0.10; 0.11) | 0.19 (0.18; 0.19) |

ii (Genetic Risk Ratio (GRR))

| Age | AD | ADHD | BD | CB | DUD | EDU | MD |
| --- | --- | --- | --- | --- | --- | --- | --- |
| 15 | 0.36 (0.35; 0.37) | 0.55 (0.53;0.56) | 0.18 (0.17;0.20) | 1.08 (1.06;1.1) | 0.89 (0.87;0.90) | 0.72 (0.71;0.74) | 0.27 (0.26;0.28) |
| 20 | 0.38 (0.37; 0.39) | 0.54 (0.53; 0.56) | 0.19 (0.18;0.20) | 1.06 (1.04;1.07) | 0.87 (0.86;0.89) | 0.71 (0.70;0.72) | 0.29 (0.28;0.30) |
| 25 | 0.39 (0.38; 0.40) | 0.54 (0.53; 0.55) | 0.19 (0.18; 0.20) | 1.03 (1.02;1.05) | 0.86 (0.85;0.88) | 0.69 (0.68;0.70) | 0.31 (0.30;0.32) |
| 30 | 0.41 (0.40; 0.42) | 0.53 (0.52; 0.54) | 0.20 (0.19; 0.20) | 1.00 (0.99; 1.01) | 0.85 (0.84;0.86) | 0.67 (0.66;0.68) | 0.33 (0.32;0.34) |
| 35 | 0.43 (0.42; 0.44) | 0.52 (0.51; 0.53) | 0.20 (0.19; 0.21) | 0.97 (0.95; 0.98) | 0.83 (0.82; 0.84) | 0.65 (0.64;0.66) | 0.36 (0.35;0.36) |
| 40 | 0.46 (0.45; 0.47) | 0.51 (0.50; 0.52) | 0.21 (0.2; 0.22) | 0.92 (0.91; 0.93) | 0.81 (0.80; 0.82) | 0.62 (0.61; 0.63) | 0.39 (0.38;0.40) |
| 45 | 0.49 (0.48; 0.50) | 0.50 (0.49; 0.51) | 0.22 (0.21; 0.23) | 0.87 (0.85; 0.88) | 0.78 (0.77; 0.08) | 0.58 (0.57; 0.59) | 0.43 (0.42; 0.44) |
| 50 | 0.53 (0.52; 0.55) | 0.49 (0.47; 0.50) | 0.23 (0.22; 0.24) | 0.79 (0.78; 0.81) | 0.75 (0.73; 0.77) | 0.53 (0.52; 0.54) | 0.48 (0.47; 0.50) |
| 55 | 0.59 (0.57; 0.61) | 0.46 (0.44; 0.49) | 0.24 (0.22; 0.26) | 0.70 (0.67; 0.72) | 0.70 (0.68; 0.73) | 0.47 (0.45; 0.48) | 0.56 (0.54; 0.58) |
| 60 | 0.67 (0.64; 0.71) | 0.43 (0.40; 0.47) | 0.26 (0.24; 0.29) | 0.56 (0.53; 0.59) | 0.64 (0.6; 0.68) | 0.37 (0.35; 0.40) | 0.66 (0.63; 0.69) |

Appendix table 7c

MD: i (mean FGRS)

| Age | AD | ADHD | AUD | BD | DEM | DUD | EDU | MD | SZ |
| --- | --- | --- | --- | --- | --- | --- | --- | --- | --- |
| 15 | 0.51 (0.49; 0.52) | 0.40 (0.39; 0.42) | 0.41 (0.39; 0.42) | 0.28 (0.26; 0.29) | 0.00 (-0.02; 0.01) | 0.46 (0.44; 0.47) | 0.24 (0.23; 0.26) | 0.60 (0.58; 0.61) | 0.17 (0.15; 0.19) |
| 20 | 0.47 (0.46; 0.48) | 0.37 (0.36; 0.39) | 0.37 (0.35; 0.38) | 0.25 (0.24; 0.26) | 0.00 (-0.01; 0.01) | 0.41 (0.40; 0.43) | 0.22 (0.21; 0.23) | 0.55 (0.54; 0.56) | 0.15 (0.14; 0.16) |
| 25 | 0.43 (0.42; 0.44) | 0.34 (0.32; 0.35) | 0.33 (0.32; 0.34) | 0.22 (0.21; 0.23) | 0.00 (-0.01; 0.01) | 0.37 (0.36; 0.38) | 0.20 (0.19; 0.21) | 0.50 (0.49; 0.51) | 0.13 (0.12; 0.14) |
| 30 | 0.40 (0.39; 0.41) | 0.30 (0.29; 0.31) | 0.29 (0.28; 0.30) | 0.20 (0.19; 0.20) | 0.00 (-0.01; 0.01) | 0.33 (0.32; 0.34) | 0.18 (0.17; 0.19) | 0.46 (0.45; 0.47) | 0.11 (0.10; 0.12) |
| 35 | 0.36 (0.35; 0.37) | 0.27 (0.26; 0.27) | 0.26 (0.25; 0.26) | 0.17 (0.16; 0.18) | 0.01 (0.00; 0.01) | 0.28 (0.27; 0.29) | 0.16 (0.15; 0.16) | 0.41 (0.40; 0.42) | 0.09 (0.08; 0.10) |
| 40 | 0.32 (0.32; 0.33) | 0.23 (0.23; 0.24) | 0.22 (0.21; 0.22) | 0.14 (0.14; 0.15) | 0.01 (0.00; 0.01) | 0.24 (0.23; 0.24) | 0.14 (0.13; 0.14) | 0.36 (0.36; 0.37) | 0.07 (0.06; 0.07) |
| 45 | 0.28 (0.28; 0.29) | 0.20 (0.19; 0.20) | 0.18 (0.18; 0.18) | 0.11 (0.11; 0.12) | 0.01 (0.01; 0.01) | 0.19 (0.19; 0.20) | 0.12 (0.11; 0.12) | 0.32 (0.31; 0.32) | 0.05 (0.05; 0.05) |
| 50 | 0.25 (0.24; 0.25) | 0.16 (0.16; 0.17) | 0.14 (0.14; 0.15) | 0.09 (0.08; 0.09) | 0.01 (0.01; 0.02) | 0.15 (0.15; 0.15) | 0.10 (0.09; 0.10) | 0.27 (0.27; 0.27) | 0.03 (0.03; 0.03) |
| 55 | 0.21 (0.21; 0.21) | 0.13 (0.12; 0.13) | 0.11 (0.10; 0.11) | 0.06 (0.06; 0.06) | 0.02 (0.01; 0.02) | 0.11 (0.10; 0.11) | 0.07 (0.07; 0.08) | 0.22 (0.22; 0.23) | 0.01 (0.01; 0.01) |
| 60 | 0.17 (0.17; 0.18) | 0.09 (0.09; 0.10) | 0.07 (0.06; 0.07) | 0.03 (0.03; 0.04) | 0.02 (0.01; 0.02) | 0.06 (0.06; 0.07) | 0.05 (0.05; 0.06) | 0.18 (0.17; 0.18) | -0.01 (-0.02; -0.01) |

ii (Genetic Risk Ratio (GRR))

| Age | AD | ADHD | AUD | BD | DEM | DUD | EDU | SZ |
| --- | --- | --- | --- | --- | --- | --- | --- | --- |
| 15 | 0.85 (0.82; 0.89) | 0.68 (0.64; 0.71) | 0.68 (0.65; 0.71) | 0.46 (0.43; 0.49) | -0.01 (0.02; -0.03) | 0.77 (0.73; 0.80) | 0.41 (0.38; 0.43) | 0.29 (0.26; 0.31) |
| 20 | 0.86 (0.82; 0.89) | 0.67 (0.64; 0.71) | 0.67 (0.64; 0.70) | 0.45 (0.43; 0.48) | 0.00 (0.02; -0.03) | 0.75 (0.72; 0.78) | 0.40 (0.38; 0.43) | 0.27 (0.25; 0.30) |
| 25 | 0.86 (0.83; 0.89) | 0.67 (0.64; 0.70) | 0.66 (0.63; 0.69) | 0.44 (0.42; 0.47) | 0.00 (0.02; -0.02) | 0.73 (0.70; 0.76) | 0.40 (0.37; 0.42) | 0.26 (0.24; 0.28) |
| 30 | 0.87 (0.84; 0.89) | 0.66 (0.63; 0.69) | 0.64 (0.62; 0.67) | 0.43 (0.40; 0.45) | 0.01 (0.03; -0.01) | 0.71 (0.69; 0.74) | 0.39 (0.37; 0.41) | 0.24 (0.22; 0.26) |
| 35 | 0.87 (0.85; 0.90) | 0.65 (0.63; 0.67) | 0.62 (0.60; 0.64) | 0.41 (0.39; 0.43) | 0.01 (0.03; 0.00) | 0.69 (0.66; 0.71) | 0.38 (0.37; 0.40) | 0.22 (0.20; 0.24) |
| 40 | 0.88 (0.86; 0.90) | 0.64 (0.62; 0.66) | 0.60 (0.58; 0.62) | 0.39 (0.37; 0.40) | 0.02 (0.04; 0.01) | 0.65 (0.63; 0.67) | 0.38 (0.36; 0.39) | 0.19 (0.18; 0.21) |
| 45 | 0.90 (0.88; 0.91) | 0.62 (0.61; 0.64) | 0.57 (0.56; 0.58) | 0.36 (0.35; 0.37) | 0.03 (0.04; 0.02) | 0.61 (0.60; 0.62) | 0.37 (0.35; 0.38) | 0.16 (0.14; 0.17) |
| 50 | 0.91 (0.9; 0.93) | 0.60 (0.59; 0.61) | 0.53 (0.52; 0.54) | 0.32 (0.31; 0.33) | 0.05 (0.06; 0.04) | 0.55 (0.54; 0.56) | 0.35 (0.34; 0.36) | 0.11 (0.10; 0.12) |
| 55 | 0.94 (0.91; 0.96) | 0.57 (0.55; 0.59) | 0.47 (0.45; 0.49) | 0.26 (0.25; 0.28) | 0.07 (0.08; 0.05) | 0.47 (0.45; 0.49) | 0.33 (0.31; 0.35) | 0.04 (0.02; 0.06) |
| 60 | 0.97 (0.93; 1.01) | 0.53 (0.49; 0.56) | 0.38 (0.35; 0.41) | 0.18 (0.15; 0.21) | 0.10 (0.13; 0.07) | 0.34 (0.31; 0.38) | 0.30 (0.27; 0.33) | -0.06 (-0.09; -0.03) |

Appendix table 7d BD:

i (mean FGRS)

| Age | AD | ADHD | AUD | BD | DEM | DUD | EDU | MD | ONAP | SZ |
| --- | --- | --- | --- | --- | --- | --- | --- | --- | --- | --- |
| 15 | 0.10  (0.07; 0.13) | 0.08  (0.05; 0.12) | 0.19  (0.16; 0.22) | 0.92  (0.88; 0.97) | 0.06  (0.03; 0.08) | 0.21  (0.18; 0.24) | -0.07  (-0.1; -0.05) | 0.21  (0.18; 0.24) | 0.68  (0.64; 0.72) | 0.50  (0.47; 0.54) |
| 20 | 0.13  (0.11; 0.16) | 0.11  (0.08; 0.14) | 0.20  (0.17; 0.22) | 0.87  (0.83; 0.91) | 0.05  (0.03; 0.07) | 0.22  (0.19; 0.24) | -0.06  (-0.08; -0.04) | 0.24  (0.21; 0.26) | 0.61  (0.58; 0.64) | 0.45  (0.42; 0.48) |
| 25 | 0.17  (0.15; 0.19) | 0.14  (0.11; 0.16) | 0.20  (0.18; 0.22) | 0.82  (0.79; 0.85) | 0.04  (0.03; 0.06) | 0.23  (0.20; 0.25) | -0.04  (-0.06; -0.03) | 0.26  (0.24; 0.28) | 0.55  (0.52; 0.57) | 0.40  (0.37; 0.43) |
| 30 | 0.20  (0.18; 0.21) | 0.16  (0.14; 0.18) | 0.21  (0.19; 0.23) | 0.77  (0.74; 0.8) | 0.04  (0.02; 0.05) | 0.23  (0.21; 0.25) | -0.03  (-0.04; -0.02) | 0.29  (0.27; 0.30) | 0.48  (0.46; 0.51) | 0.35  (0.33; 0.37) |
| 35 | 0.23  (0.22; 0.24) | 0.19  (0.17; 0.20) | 0.22  (0.21; 0.23) | 0.72  (0.70; 0.74) | 0.00  (0.02; 0.05) | 0.24  (0.23; 0.25) | -0.02  (-0.03; 0.00) | 0.31  (0.30; 0.33) | 0.42  (0.40; 0.44) | 0.30  (0.28; 0.31) |
| 40 | 0.26  (0.25; 0.27) | 0.21  (0.20; 0.22) | 0.23  (0.22; 0.24) | 0.67  (0.65; 0.68) | 0.00  (0.02; 0.04) | 0.25  (0.24; 0.26) | 0.00  (-0.01; 0.01) | 0.34  (0.33; 0.35) | 0.36  (0.34; 0.37) | 0.24  (0.23; 0.26) |
| 45 | 0.29  (0.28; 0.30) | 0.24  (0.23; 0.25) | 0.23  (0.22; 0.24) | 0.62  (0.60; 0.63) | 0.00  (0.01; 0.03) | 0.26  (0.24; 0.27) | 0.01  (0.01; 0.02) | 0.36  (0.35; 0.37) | 0.29  (0.28; 0.30) | 0.19  (0.18; 0.2,) |
| 50 | 0.32  (0.31; 0.34) | 0.26  (0.25; 0.28) | 0.24  (0.23; 0.25) | 0.56  (0.55; 0.58) | 0.00  (0.01; 0.03) | 0.26  (0.25; 0.28) | 0.03  (0.02; 0.04) | 0.39  (0.38; 0.40) | 0.23  (0.21; 0.24) | 0.14  (0.12; 0.15) |
| 55 | 0.36  (0.34; 0.37) | 0.29  (0.27; 0.30) | 0.25  (0.23; 0.26) | 0.51  (0.49; 0.54) | 0.01  (0.00; 0.03) | 0.27  (0.25; 0.29) | 0.04  (0.03; 0.06) | 0.41  (0.40; 0.43) | 0.16  (0.14; 0.18) | 0.09  (0.07; 0.11) |
| 60 | 0.39  (0.37; 0.41) | 0.31  (0.29; 0.33) | 0.25  (0.24; 0.27) | 0.46  (0.43; 0.49) | 0.01  (-0.01; 0.02) | 0.28  (0.26; 0.30) | 0.06  (0.04; 0.07) | 0.44 (0.42; 0.46) | 0.10  (0.07; 0.12) | 0.04  (0.01; 0.06) |

ii (Genetic Risk Ratio (GRR))

| Age | AD | ADHD | AUD | DEM | DUD | EDU | MD | ONAP | SZ |
| --- | --- | --- | --- | --- | --- | --- | --- | --- | --- |
| 15 | 0.11 (0.08; 0.14) | 0.09 (0.06; 0.13) | 0.20 (0.17; 0.24) | 0.06 (0.03; 0.09) | 0.23 (0.19; 0.26) | -0.08 (-0.05; -0.11) | 0.23 (0.20; 0.26) | 0.73 (0.68; 0.79) | 0.55 (0.50; 0.60) |
| 20 | 0.15 (0.13; 0.18) | 0.13 (0.09; 0.16) | 0.23 (0.19; 0.26) | 0.06 (0.03; 0.08) | 0.25 (0.22; 0.28) | -0.07 (-0.04; -0.09) | 0.27 (0.24; 0.30) | 0.70 (0.65; 0.75) | 0.52 (0.47; 0.56) |
| 25 | 0.20 (0.18; 0.23) | 0.17 (0.14; 0.19) | 0.25 (0.22; 0.28) | 0.05 (0.03; 0.08) | 0.27 (0.24; 0.30) | -0.05 (-0.03; -0.08) | 0.32 (0.29; 0.35) | 0.67 (0.63; 0.71) | 0.49 (0.45; 0.53) |
| 30 | 0.26 (0.23; 0.28) | 0.21 (0.18; 0.23) | 0.27 (0.25; 0.30) | 0.05 (0.03; 0.07) | 0.30 (0.28; 0.33) | -0.04 (-0.02; -0.06) | 0.37 (0.35; 0.40) | 0.63 (0.59; 0.66) | 0.45 (0.42; 0.48) |
| 35 | 0.32 (0.30; 0.34) | 0.26 (0.24; 0.28) | 0.30 (0.28; 0.32) | 0.05 (0.03; 0.06) | 0.33 (0.31; 0.36) | -0.02 (-0.01; -0.04) | 0.44 (0.41; 0.46) | 0.58 (0.56; 0.61) | 0.41 (0.39; 0.44) |
| 40 | 0.39 (0.37; 0.41) | 0.32 (0.30; 0.34) | 0.34 (0.32; 0.36) | 0.04 (0.03; 0.06) | 0.37 (0.35; 0.39) | 0.00 (0.01; -0.01) | 0.51 (0.49; 0.53) | 0.53 (0.51; 0.56) | 0.37 (0.34; 0.39) |
| 45 | 0.48 (0.46; 0.50) | 0.39 (0.36; 0.41) | 0.38 (0.36; 0.40) | 0.04 (0.02; 0.05) | 0.42 (0.39; 0.44) | 0.02 (0.04; 0.01) | 0.59 (0.57; 0.61) | 0.47 (0.45; 0.50) | 0.31 (0.29; 0.33) |
| 50 | 0.58 (0.55; 0.60) | 0.47 (0.44; 0.49) | 0.43 (0.40; 0.45) | 0.03 (0.01; 0.05) | 0.47 (0.44; 0.49) | 0.05 (0.07; 0.03) | 0.69 (0.66; 0.72) | 0.40 (0.37; 0.43) | 0.25 (0.22; 0.28) |
| 55 | 0.70 (0.65; 0.74) | 0.56 (0.52; 0.60) | 0.48 (0.45; 0.52) | 0.02 (0.00; 0.05) | 0.53 (0.49; 0.57) | 0.08 (0.11; 0.06) | 0.81 (0.76; 0.86) | 0.32 (0.28; 0.35) | 0.17 (0.13; 0.21) |
| 60 | 0.84 (0.78; 0.91) | 0.68 (0.62; 0.74) | 0.55 (0.50; 0.61) | 0.02 (-0.02; 0.05) | 0.6 0(0.55; 0.66) | 0.12 (0.16; 0.09) | 0.95 (0.88; 1.03) | 0.21 (0.16; 0.26) | 0.08 (0.02; 0.13) |

Appendix table 7e SZ:

i (mean FGRS)

| Age | AD | ADHD | ASD | AUD | BD | DEM | DUD | EDU | MD | ONAP | SZ |
| --- | --- | --- | --- | --- | --- | --- | --- | --- | --- | --- | --- |
| 15 | -0.02  (-0.05; 0.01) | 0.02  (-0.01; 0.04) | 0.3  (0.26; 0.34) | 0.20  (0.17; 0.23) | 0.28  (0.25; 0.32) | 0.02  (-0.01; 0.05) | 0.14  (0.11; 0.17) | -0.06  (-0.08; -0.03) | 0.02  (-0.01; 0.04) | 0.66  (0.61; 0.71) | 1.01  (0.94; 1.08) |
| 20 | 0.00  (-0.02; 0.03) | 0.03  (0.01; 0.05) | 0.28  (0.25; 0.31) | 0.20  (0.17; 0.22) | 0.27  (0.24; 0.30) | 0.02  (0.00; 0.05) | 0.15  (0.12; 0.17) | -0.04  (-0.06; -0.02) | 0.03  (0.01; 0.05) | 0.64  (0.6; 0.68) | 0.94  (0.88; 1.00) |
| 25 | 0.03  (0.01; 0.04) | 0.04  (0.02; 0.05) | 0.26  (0.24; 0.29) | 0.20  (0.18; 0.22) | 0.26  (0.24; 0.28) | 0.03  (0.01; 0.05) | 0.16  (0.14; 0.18) | -0.02  (-0.03; 0.00) | 0.04  (0.03; 0.06) | 0.62  (0.58; 0.65) | 0.88  (0.83; 0.92) |
| 30 | 0.05  (0.04; 0.07) | 0.05  (0.03; 0.06) | 0.25  (0.23; 0.27) | 0.20  (0.18; 0.22) | 0.25  (0.23; 0.27) | 0.03  (0.02; 0.05) | 0.17  (0.15; 0.19) | 0.00  (-0.01; 0.02) | 0.06  (0.04; 0.07) | 0.59  (0.57; 0.62) | 0.81  (0.77; 0.85) |
| 35 | 0.07  (0.06; 0.09) | 0.06  (0.04; 0.07) | 0.23  (0.21; 0.25) | 0.20  (0.19; 0.22) | 0.24  (0.22; 0.25) | 0.03  (0.02; 0.05) | 0.18  (0.17; 0.2) | 0.02  (0.01; 0.04) | 0.07  (0.06; 0.08) | 0.57  (0.55; 0.6) | 0.75  (0.71; 0.78) |
| 40 | 0.10  (0.08; 0.11) | 0.07  (0.05; 0.08) | 0.21  (0.19; 0.23) | 0.20  (0.19; 0.22) | 0.22  (0.21; 0.24) | 0.04  (0.02; 0.05) | 0.19  (0.18; 0.21) | 0.04  (0.03; 0.06) | 0.09  (0.07; 0.1) | 0.55  (0.52; 0.58) | 0.68  (0.64; 0.72) |
| 45 | 0.12  (0.1; 0.14) | 0.08  (0.06; 0.09) | 0.19  (0.17; 0.21) | 0.20  (0.19; 0.22) | 0.21  (0.19; 0.23) | 0.04  (0.02; 0.05) | 0.20  (0.19; 0.22) | 0.06  (0.05; 0.08) | 0.10  (0.08; 0.12) | 0.53  (0.5; 0.56) | 0.61  (0.57; 0.66) |
| 50 | 0.14  (0.12; 0.16) | 0.09  (0.07; 0.10) | 0.17  (0.15; 0.2) | 0.21  (0.18; 0.23) | 0.2  (0.17; 0.23) | 0.04  (0.02; 0.06) | 0.21  (0.19; 0.24) | 0.09  (0.07; 0.10) | 0.11  (0.09; 0.13) | 0.50  (0.47; 0.54) | 0.55  (0.50; 0.60) |
| 55 | 0.17  (0.14; 0.19) | 0.10  (0.07; 0.12) | 0.16  (0.12; 0.19) | 0.21  (0.18; 0.23) | 0.19  (0.16; 0.22) | 0.04  (0.02; 0.07) | 0.23  (0.2; 0.25) | 0.11  (0.08; 0.13) | 0.13  (0.1; 0.15) | 0.48  (0.44; 0.53) | 0.48  (0.42; 0.55) |
| 60 | 0.19  (0.16; 0.22) | 0.10  (0.08; 0.13) | 0.14  (0.10; 0.18) | 0.21  (0.17; 0.24) | 0.18  (0.14; 0.22) | 0.05  (0.02; 0.08) | 0.24  (0.20; 0.27) | 0.13  (0.10; 0.16) | 0.14  (0.11; 0.17) | 0.46  (0.41; 0.52) | 0.42  (0.34; 0.49) |

ii (Genetic Risk Ratio (GRR))

| Age | AD | ADHD | ASD | AUD | BD | DEM | DUD | EDU | MD | ONAP |
| --- | --- | --- | --- | --- | --- | --- | --- | --- | --- | --- |
| 15 | -0.02  (0.01; -0.05) | 0.02  (-0.01;0.04) | 0.30 (0.25;0.34) | 0.20 (0.16;0.23) | 0.28 (0.24;0.32) | 0.02  (-0.01;0.05) | 0.13 (0.1;0.17) | -0.06  (-0.03; -0.08) | 0.02  (-0.01; 0.04) | 0.66  (0.59; 0.72) |
| 20 | 0.00  (0.03; -0.02) | 0.03  (0.01; 0.05) | 0.30 (0.26;0.34) | 0.21 (0.18;0.24) | 0.29 (0.25;0.32) | 0.03  (0.00; 0.05) | 0.16 (0.13;0.18) | -0.04  (-0.02; -0.06) | 0.03  (0.01; 0.06) | 0.68  (0.62; 0.74) |
| 25 | 0.03  (0.05; 0.01) | 0.04  (0.02; 0.06) | 0.30  (0.27; 0.33) | 0.23 (0.2;0.25) | 0.30 (0.26;0.33) | 0.03 (0.01;0.05) | 0.18 (0.16;0.21) | -0.02  (0; -0.04) | 0.05  (0.03; 0.07) | 0.70  (0.65; 0.76) |
| 30 | 0.06  (0.08; 0.04) | 0.06  (0.04; 0.07) | 0.30  (0.27; 0.33) | 0.25  (0.22; 0.27) | 0.31 (0.28;0.33) | 0.04 (0.02;0.06) | 0.21 (0.19;0.23) | 0.00  (0.02; -0.01) | 0.07  (0.05; 0.09) | 0.73  (0.68; 0.78) |
| 35 | 0.10  (0.12; 0.08) | 0.08  (0.06; 0.09) | 0.31  (0.28; 0.33) | 0.27  (0.25; 0.29) | 0.32  (0.29; 0.34) | 0.04 (0.03;0.06) | 0.24 (0.22;0.27) | 0.03  (0.05; 0.01) | 0.10  (0.08; 0.11) | 0.77  (0.72; 0.82) |
| 40 | 0.14  (0.16; 0.12) | 0.10  (0.08; 0.12) | 0.31  (0.28; 0.34) | 0.30  (0.27; 0.33) | 0.33  (0.3; 0.36) | 0.05  (0.03; 0.07) | 0.28 (0.26;0.31) | 0.07  (0.09; 0.05) | 0.13  (0.11; 0.15) | 0.81  (0.75; 0.87) |
| 45 | 0.20  (0.23; 0.17) | 0.12  (0.1; 0.15) | 0.31  (0.27; 0.36) | 0.33  (0.29; 0.37) | 0.35  (0.3; 0.39) | 0.06  (0.03; 0.09) | 0.33  (0.29; 0.37) | 0.11  (0.13; 0.08) | 0.16  (0.13; 0.19) | 0.86  (0.78; 0.94) |
| 50 | 0.26  (0.31; 0.22) | 0.16  (0.12; 0.19) | 0.32  (0.26; 0.38) | 0.37  (0.32; 0.43) | 0.37  (0.31; 0.43) | 0.07  (0.04; 0.11) | 0.39  (0.34; 0.45) | 0.16  (0.19; 0.12) | 0.21  (0.16; 0.25) | 0.92  (0.81; 1.03) |
| 55 | 0.35  (0.42; 0.28) | 0.2  (0.14; 0.25) | 0.32  (0.24; 0.41) | 0.43  (0.35; 0.51) | 0.39  (0.31; 0.48) | 0.09  (0.04; 0.14) | 0.47  (0.38; 0.55) | 0.22  (0.28; 0.16) | 0.26  (0.2; 0.32) | 1.00  (0.83; 1.16) |
| 60 | 0.46  (0.57; 0.35) | 0.25  (0.17; 0.33) | 0.33  (0.22; 0.45) | 0.50  (0.37; 0.62) | 0.43  (0.3; 0.55) | 0.11  (0.03; 0.19) | 0.57  (0.44; 0.7) | 0.30  (0.39; 0.21) | 0.34  (0.24; 0.43) | 1.10  (0.86; 1.35) |

Table 8 Patterns of Comorbidity in our Primary Diagnoses

|  | DUD | AUD | SZ | BD | MD* | Unique cases |
| --- | --- | --- | --- | --- | --- | --- |
| DUD | - | 38.1% | 2.5% | 7.5% | 35.4% | 83,090 (36.9%) |
| AUD | 25.8% |  | 1.8% | 5.0% | 29.8% | 170,955 (51.4%) |
| SZ | 21.7% | 23.4% | - | 15.0% | 17.4% | 12,098 (47.0%) |
| BD | 23.5% | 23.2% | 5.4% | - | - | 44,131 (61.2%) |
| MD | 9.9% | 12.3% | 0.6% | - | - | 658,235 (81.7%) |
|  |  |  |  |  |  |  |

Figure 1a - AUD: No DUD registrations (74.1%% of the original sample) and No MD registrations (66.7% of the original sample)


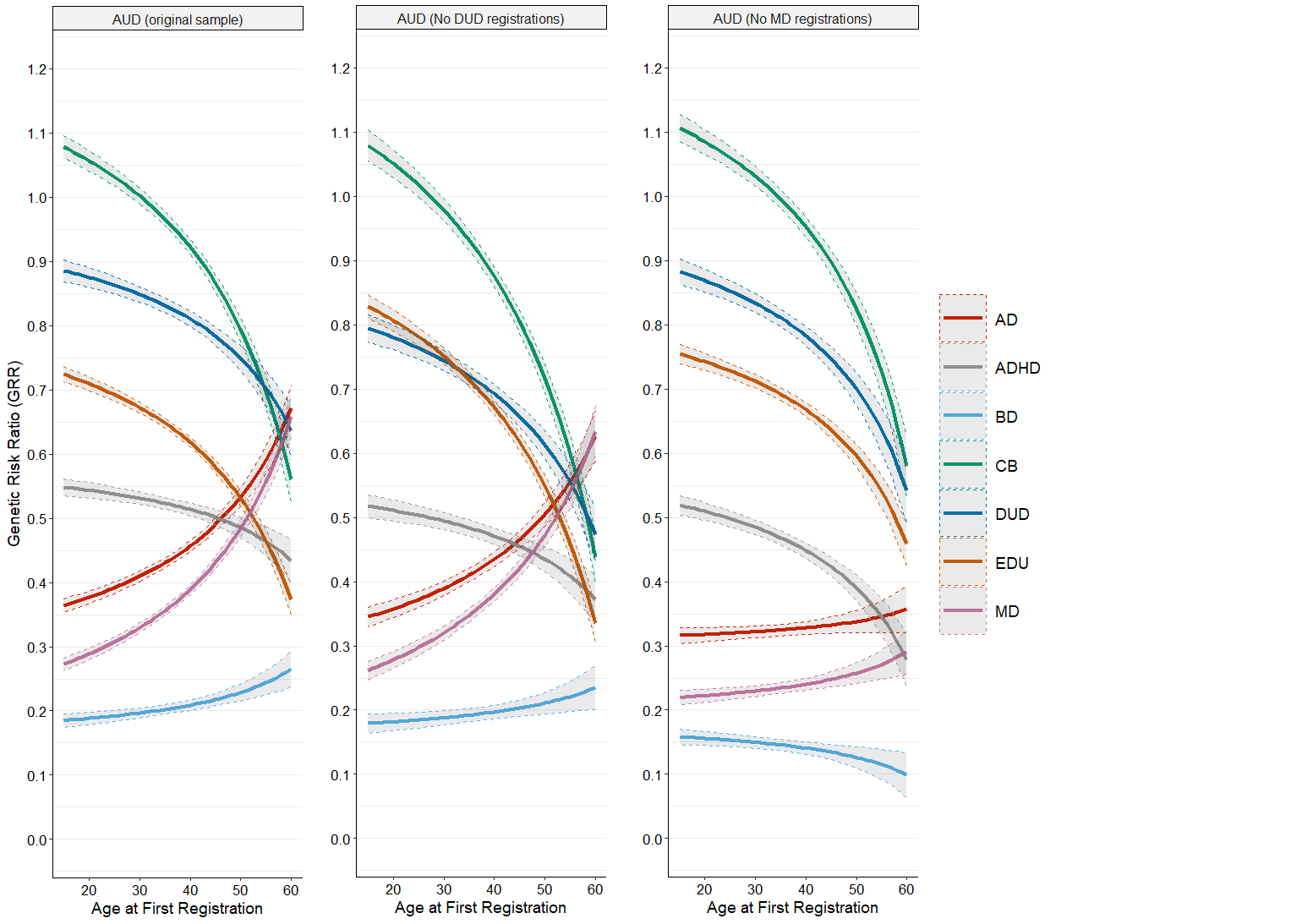


Figure 1b DUD: No AUD registrations (61.9% of the original sample) and No MD registrations (59.0% of the original sample)


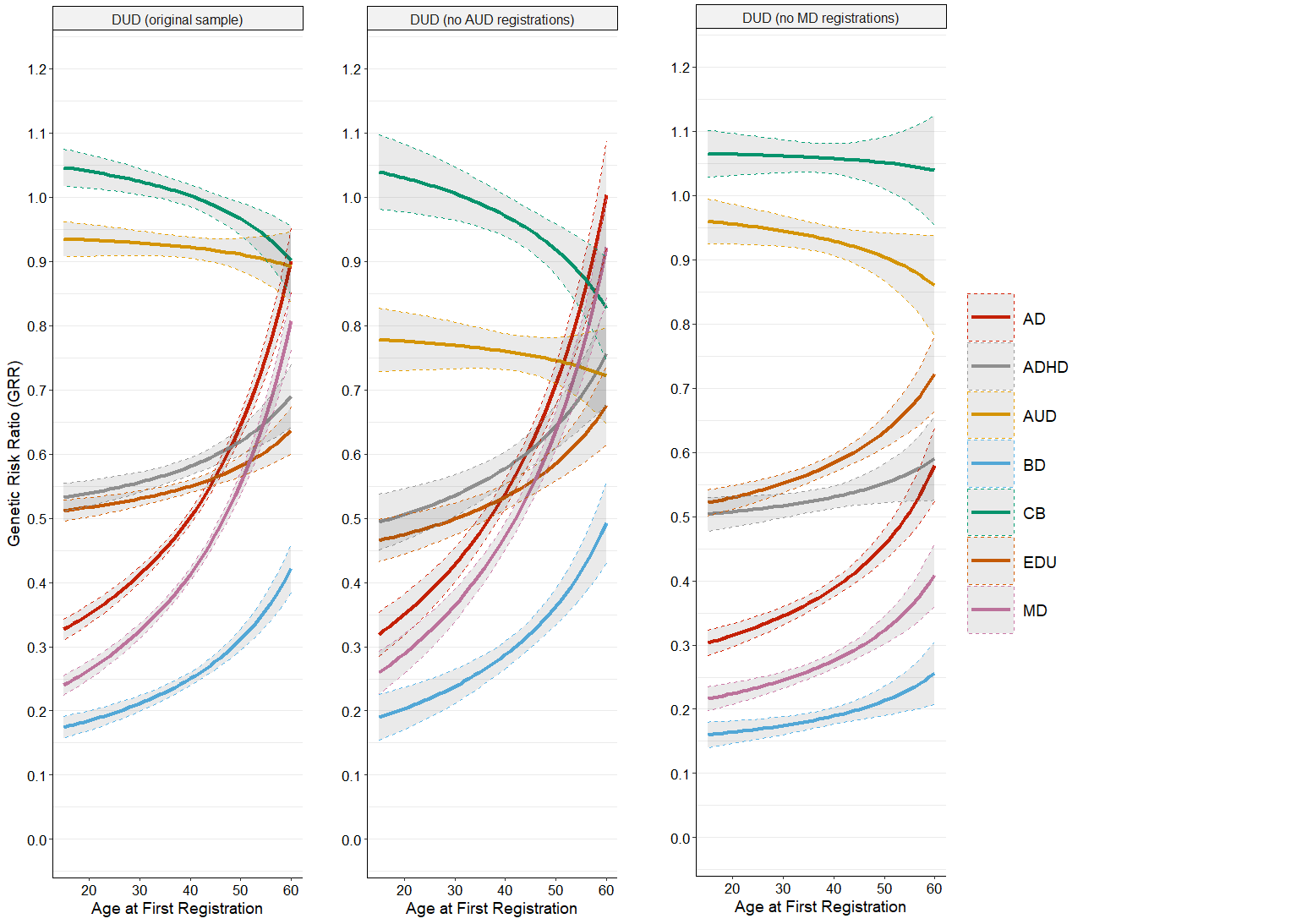


Figure 1c - MD: No DUD registrations (90.0% of the original sample)


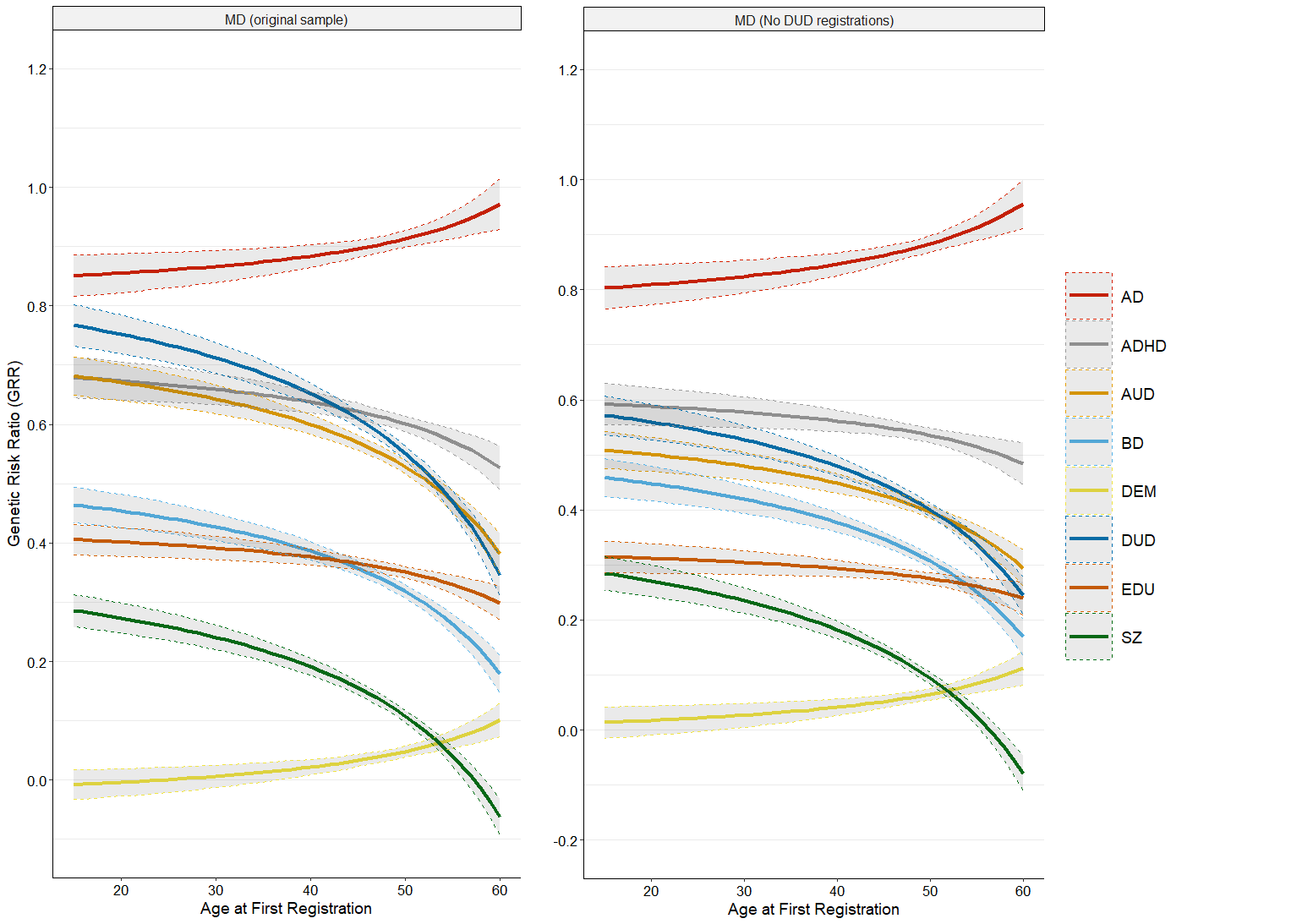


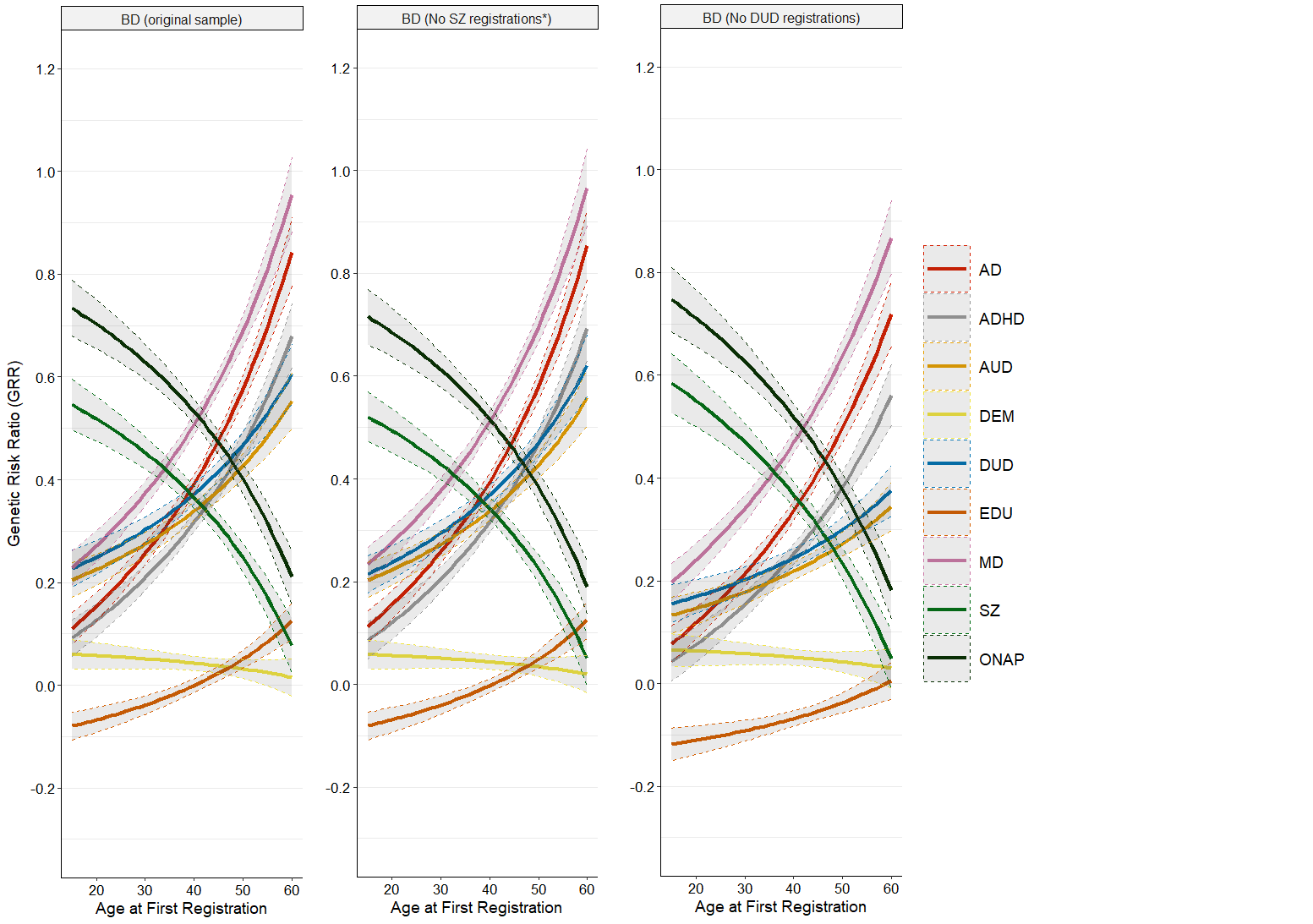
Figure 1d - BD: No SZ registrations (based on hierarchy of registrations) (97.7% of the original sample) and No DUD registrations (76.4% of the original sample)

Figure 1e - SZ: No BD registrations (based on hierarchy of registrations) (91.2% of the original sample)


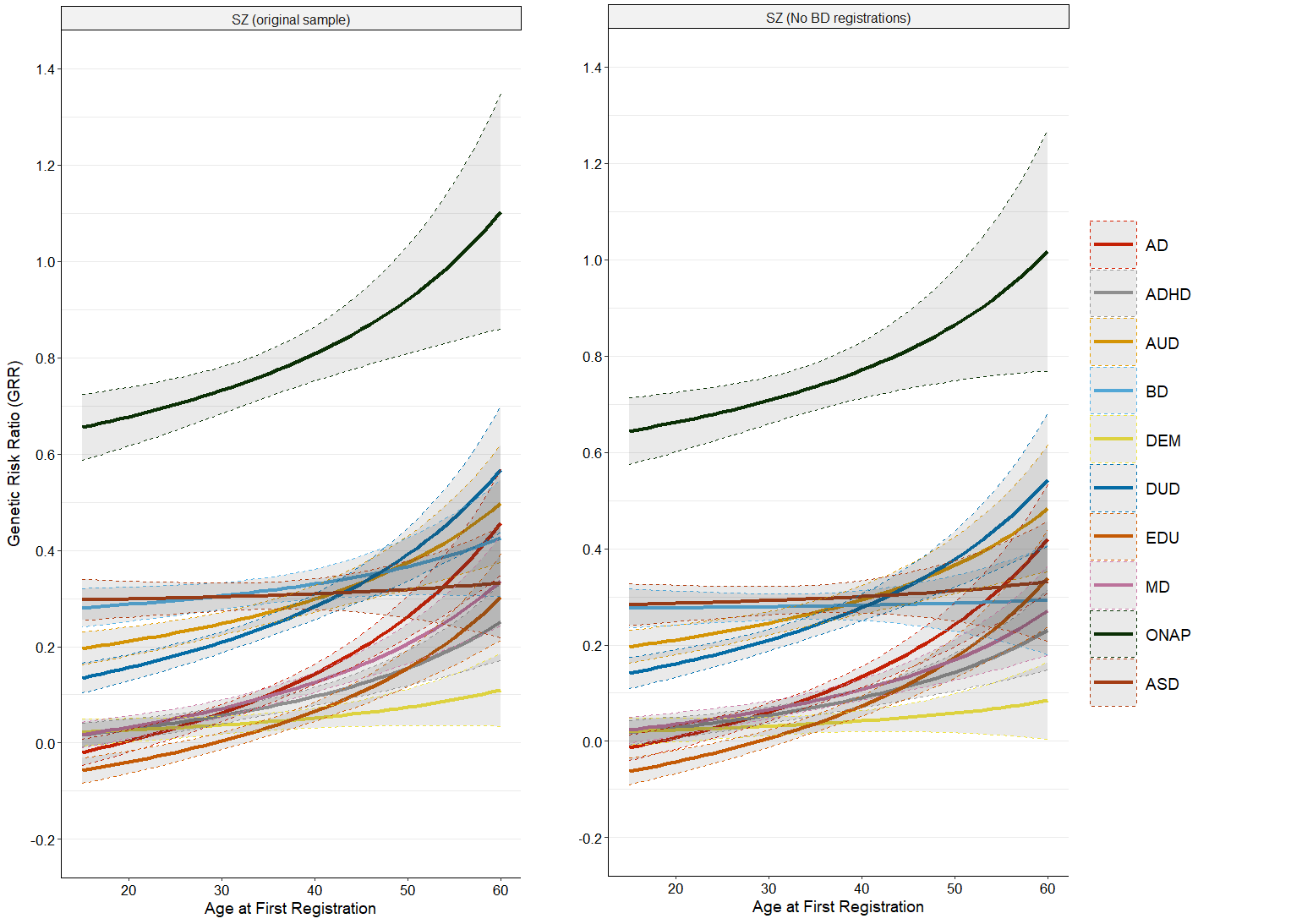


Figure 2a Different Cohorts AUD


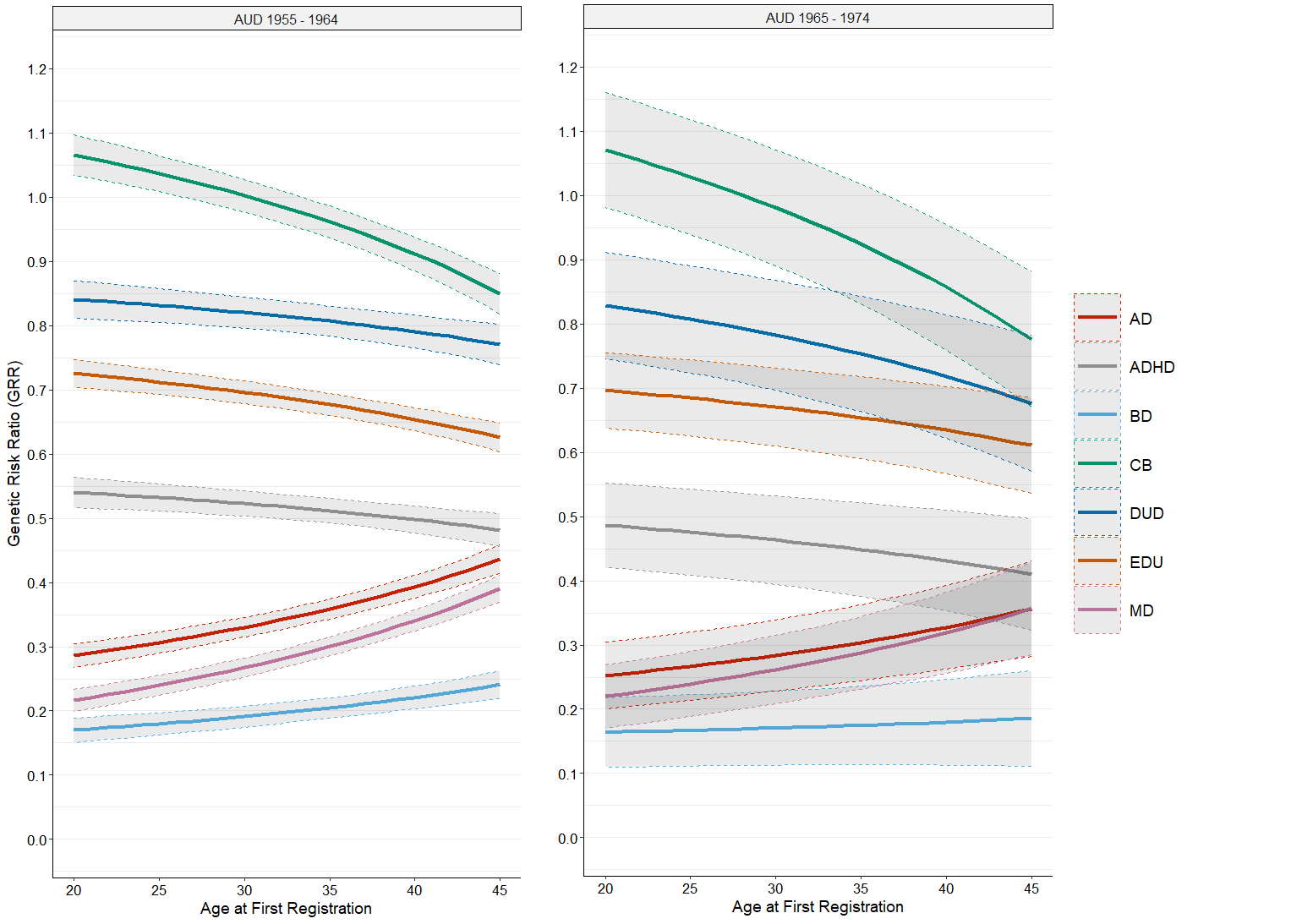


Figure 2b Different Cohorts DUD


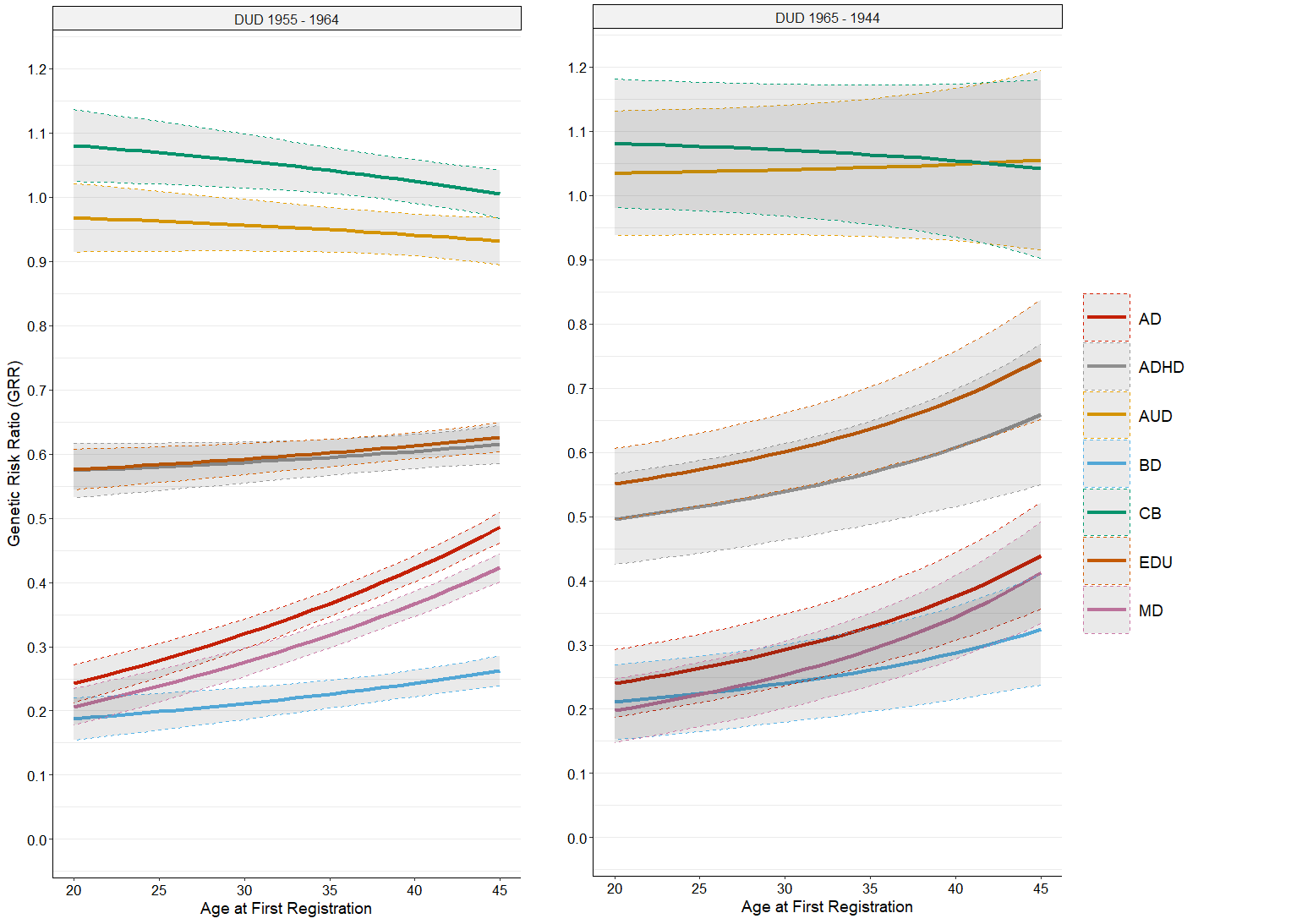


Figure 2c Different Cohorts MD


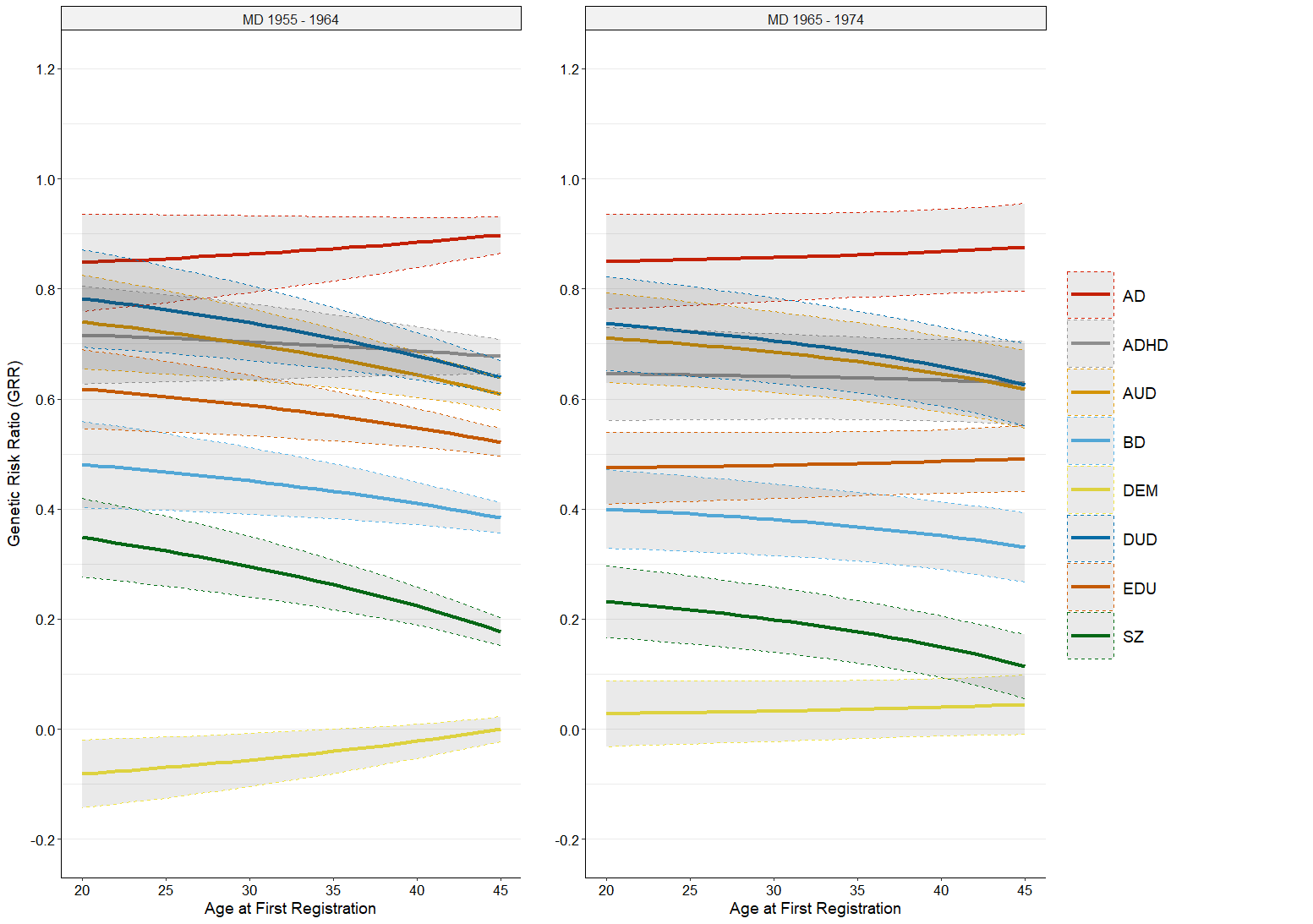


Figure 2d Different Cohorts BD


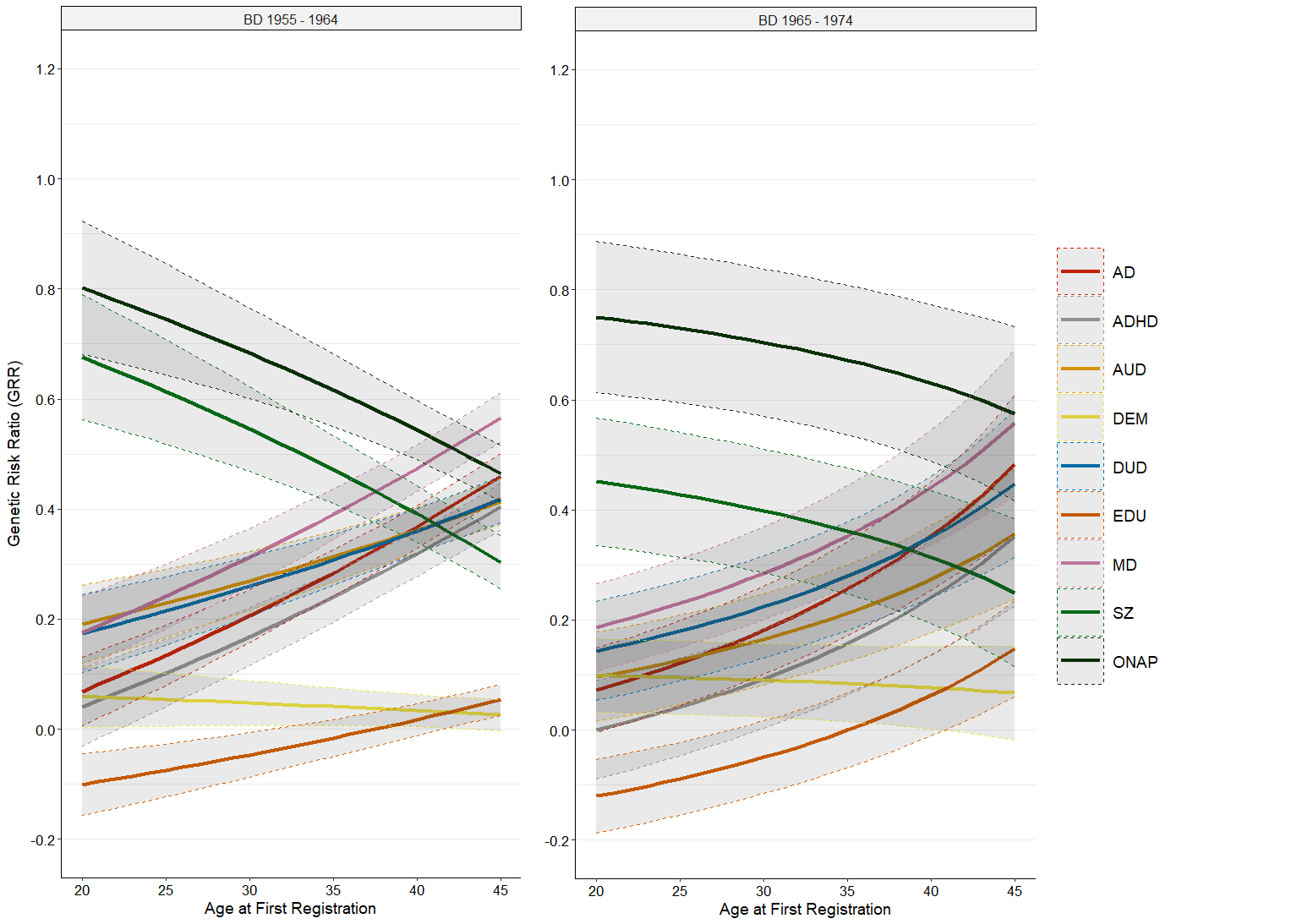


Figure 2e Different Cohorts SZ


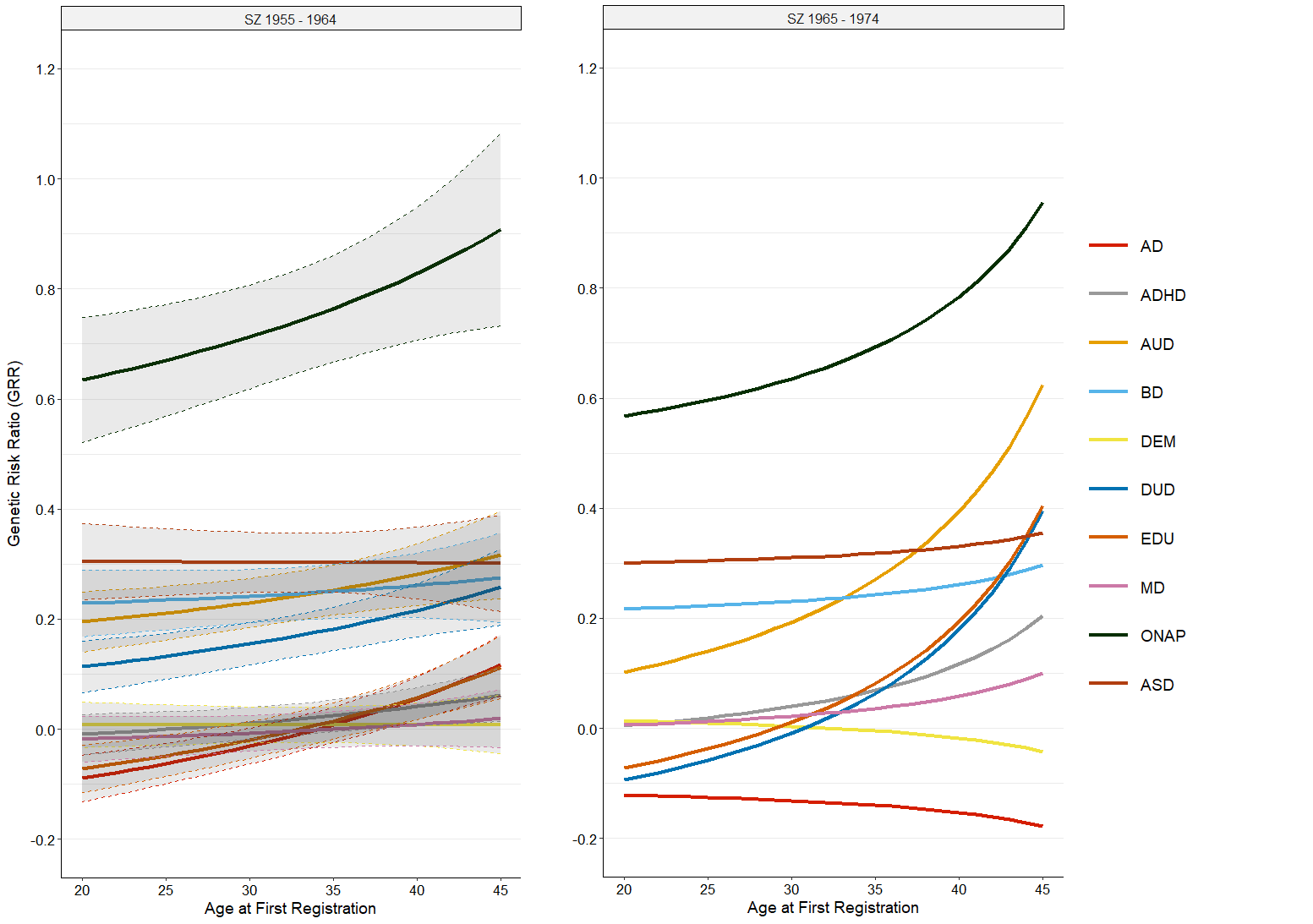

Supplement: Kendler et al. supplementary material [file S0033291724002630sup001.docx]
